# Dynamic molecular regulation of salt stress responses in maize (*Zea mays* L.) seedlings

**DOI:** 10.3389/fpls.2025.1535943

**Published:** 2025-02-25

**Authors:** Atikaimu Maimaiti, Wei Gu, Diansi Yu, Yuan Guan, Jingtao Qu, Tao Qin, Hui Wang, Jiaojiao Ren, Hongjian Zheng, Penghao Wu

**Affiliations:** ^1^ College of Agriculture, Xinjiang Agricultural University, Urumqi, China; ^2^ Crop Breeding, Cultivation Research Institution/Centro Internacional de Mejoramientode Maizy Trigo (CIMMYT)-China Specialty Maize Research Center, Shanghai Engineering Research Center of Specialty Maize, Shanghai Key Laboratory of Agricultural Genetics and Breeding, Shanghai Academy of Agricultural Sciences, Shanghai, China

**Keywords:** maize, salt stress, transcriptome analysis, DEGs, WGCNA

## Abstract

**Introduction:**

Maize ranks among the most essential crops globally, yet its growth and yield are significantly hindered by salt stress, posing challenges to agricultural productivity. To utilize saline-alkali soils more effectively and enrich maize germplasm resources, identifying salt-tolerant genes in maize is essential.

**Methods:**

In this study, we used a salt-tolerant maize inbred line, SPL02, and a salt-sensitive maize inbred line, Mo17. We treated both lines with 180 mmol/L sodium chloride (NaCl) for 0 days, 3 days, 6 days, and 9 days at the three-leaf growth stage (V3). Through comprehensive morphological, physiological, and transcriptomic analyses, we assessed salt stress effects and identified hub genes and pathways associated with salt tolerance.

**Results:**

Our analysis identified 25,383 expressed genes, with substantial differences in gene expression patterns across the salt treatment stages. We found 8,971 differentially expressed genes (DEGs)—7,111 unique to SPL02 and 4,791 unique to Mo17—indicating dynamic gene expression changes under salt stress. In SPL02, the DEGs are primarily associated with the MAPK signaling pathway, phenylpropanoid biosynthesis, and hormone signaling under salt treatment conditions. In Mo17, salt stress responses are primarily mediated through the abscisic acid-activated signaling pathway and hormone response. Additionally, our weighted gene co-expression network analysis (WGCNA) pinpointed five hub genes that likely play central roles in mediating salt tolerance. These genes are associated with functions including phosphate import ATP-binding protein, glycosyltransferase, and WRKY transcription factors.

**Discussion:**

This study offers valuable insights into the complex regulatory networks governing the maize response to salt stress and identifies five hub genes and pathways for further investigation. These findings contribute valuable knowledge for enhancing agricultural resilience and sustainability in saline-affected environments.

## Introduction

Salt stress is a major abiotic constraint across the globe that adversely affects the growth, development, and yield of crops (Liu and Wang, 2021). Soil salinity has affected more than 7% of the total land area (1,125 million hectares) worldwide (Qu et al., 2024). A recent report by the Food and Agriculture Organization of the United Nations (FAO) in 2021 claims that the global area of agricultural land exceeds 833 million hectares, which is expected to further rise (Zhou and Li, 2024). It is estimated that more than half of arable land worldwide will be salinized by 2050 (Pincay and Cantos, 2024). China has been the country with the largest area of salinity-affected soils, with 211 million hectares (Zhou and Li, 2024). Salt stress has severely impeded agricultural growth as salinized land continues to expand. Salt stress is a significant barrier to achieving global food security (Farooq et al., 2015).

Plants grown under salt stress face a range of challenges that impact their growth and survival. This stress leads to osmotic stress, ionic toxicity, and complex secondary effects (Zhu, 2016). However, osmotic stress and sodium ion (Na^+^) toxicity are considered the principal components of the plant (Munns, 2002). Sodium chloride (NaCl) is widely studied among researchers on osmotic, ionic, and oxidative stress under saline conditions due to its high solubility and ubiquitous distribution (Zhu et al., 2023). Plants in high-salinity environments will cause osmotic stress by reducing water absorption, and plant roots will also sustain ion toxicity by absorbing a large amount of Na^+^ and Cl^−^ ions (Rahman et al., 2021). These can severely impact crop yields by interfering with normal plant growth and development. To manage salt stress, plants utilize a range of adaptive mechanisms, such as limiting the amount of salt absorbed by the roots and regulating its distribution within tissues and cells to prevent toxic accumulation in the cytosol of functional leaves (Munns, 2005; Loescher et al., 2011). Understanding these molecular mechanisms is crucial for improving salt tolerance in plants, and substantial research efforts are focused on elucidating how these processes can be enhanced to boost crop resilience to saline conditions.

Maize (*Zea mays* L.) is an important C4 crop in the Poaceae family and exhibits moderate sensitivity to salt stress (Huqe et al., 2021). The seedling stage of maize is particularly vulnerable to salt stress compared to other developmental stages (Luo et al., 2021). Accumulation of salt can hinder maize growth, and high salt concentrations can be toxic, leading to decreased production (Ali Turan et al., 2009). Salt stress adversely affects maize seedlings by slowing their growth, reducing survival rates, and damaging the photosynthetic system, which impact later development and overall yield (Chen et al., 2019). Additionally, salt stress disrupts metabolic pathways, including signal transduction, energy metabolism, and hormone synthesis (Xu et al., 2021). Different genotypes of maize exhibit variable responses to salt stress, as evidenced by changes in morphology, photosynthesis, carbon metabolism, and endogenous hormone levels, which indicate varying degrees of damage. Overall, salt stress leads to ionic imbalances, increased osmotic pressure, and oxidative stress in crops. The mechanism of maize response to salt stress has been widely studied using transcriptomic profiling. The transcriptome in a narrow sense usually refers to mRNA as the object of study, which exhibits distinct spatial and temporal constraints. These constraints result in variations in gene expression across various cell types, environmental conditions, and developmental phases within the same species (Li et al., 2022b). Several studies have investigated the regulation process of different maize inbred lines to salt stress. For instance, a salt-sensitive maize inbred line, RILbro-W22, was compared with a salt-tolerant inbred line, RILpur-W22 (Wang et al., 2023). Comparisons between salt-sensitive and salt-tolerant maize inbred lines have highlighted significant differences in gene expression profiles. A total of 3,160 differentially expressed genes (DEGs) were identified, primarily enriched in processes such as redox, monomer metabolism, catalytic activity, plasma membrane functions, and metabolic regulation. Such findings underscore the effectiveness of contrasting inbred lines with varying salt tolerances to uncover the mechanisms driving salt stress resilience. In a study that examined the impact of salt stress on maize (Zhu et al., 2023), transcriptomic analysis revealed 11,074 DEGs, highlighting the plant’s robust molecular adjustments to salinity. These findings suggest that maize establishes a form of stress memory, enabling it to better cope with recurring salt stress. Key protective components, such as proline, and critical physiological processes, particularly photosynthesis, play essential roles in the development and maintenance of this adaptive memory. These mechanisms underscore the plant’s ability to enhance its resilience to challenging saline environments. Since there could be expression of different genes at different phases of salt stress, this would allow for early- and late-response gene identification. Early-response genes may be associated with signaling and early response; later genes may be linked with metabolic adaptation genes and growth recovery genes (Ashapkin et al., 2020). In addition, weighted gene co-expression network analysis (WGCNA) is a fast and efficient method for identifying functionally relevant genes within co-expression modules (Niemira et al., 2020). It has been widely applied across various crops to uncover candidate genes linked to abiotic responses (Li et al., 2022c; Wu et al., 2022; Chen et al., 2024). Salt stress is a significant challenge to maize production (Auti et al., 2023). Previous research has identified various mechanisms through which maize responds to salinity, including ion homeostasis, osmotic adjustment, and the activation of specific stress-responsive genes (Cao et al., 2023). Studies have shown that maize varieties vary widely in their salt tolerance, with some varieties exhibiting better growth and yield stability under saline conditions. A growing body of work has focused on identifying key genes and pathways involved in salt stress tolerance, such as the Salt Overly Sensitive (SOS) pathway (Li et al., 2023b), which regulates ion transport, and transcription factors like WRKYs and NACs (Singh et al., 2024; Wagan et al., 2024), which mediate stress responses. Despite these advancements, the genetic and molecular basis of salt tolerance in maize remains complex and not fully understood.

In this study, we aimed to gain further insights into the response mechanisms of maize to salt stress and to identify hub genes associated with salt tolerance. We selected two inbred maize lines, SPL02 and Mo17, and treated them with salt at the three-leaf (V3) growth stage. Through transcriptomic analysis, we investigated DEGs and their expression patterns under salt stress to reveal the pathways involved in the stress response. In addition, we integrated transcriptomic data with phenotypic indicators using WGCNA to identify coordinated gene expression patterns and identified some hub genes that may have a strong influence on maize salt stress. These findings provide a deeper understanding of the genetic and molecular pathways driving maize’s resilience to salt stress, establishing a solid theoretical foundation for molecular marker-assisted breeding and contributing to agricultural advancement by offering valuable insights into the mechanisms underlying salt tolerance, which can be applied to improve breeding strategies, enhancing resilience to saline conditions, and supporting global food security.

## Materials and methods

### Plant material and treatments

A salt-tolerant inbred line, SPL02, and a salt-sensitive inbred line, Mo17, were provided by the Shanghai Academy of Agricultural Science (Shanghai, China). Two maize inbred lines were sown in the artificial climate chamber (day/night temperature of 28°C/18°C, humidity of 55%, and 12 hours of darkness/12 hours of light) in a seedling tray equipped with vermiculite and perlite. Hoagland nutrient solution was used to maintain a water level of 2 cm (3 L). During the V3 stage, the seedlings were divided into two groups: one group was the control (CK) group, and the other group was the salt treatment (T) group. The T group was irrigated with 180 mmol/L (10.52 g/L) NaCl solution containing nutrient solution, and the CK group was irrigated with Hoagland nutrient solution, maintaining a water level of 2 cm (3 L) in the tray. Two maize inbred lines were subjected to NaCl treatment for 0 days, 3 days, 6 days, and 9 days, referred to as 0 days after treatment (DAT), 3 DAT, 6 DAT, and 9 DAT, respectively.

### Morphological and physiological characterization

The physiological and phenotypic indicators of the seedlings were determined at 0 DAT, 3 DAT, 6 DAT, and 9 DAT. The following were measured: plant height, root length, fresh weight of whole seedlings, and chlorophyll content [soil plant analysis development (SPAD)]. 1) Determination of plant height, root length, and root number: the plant height and length of the main root of seedlings were measured using a straight ruler (Chen et al., 2020). 2) Determination of fresh weight: maize seedlings were carefully removed from the pots, the vermiculite attached to the roots was rinsed off with water, and the excess water on the surface was absorbed with filter paper. The aboveground and underground parts were then weighed on a balance (Chen et al., 2020). 3) Determination of SPAD: leaves from the bottom of the plant were counted to identify the second fully expanded leaf. The SPAD value was measured using a SPAD meter (SPAD-502, Konica Minolta, Tokyo, Japan) on the selected leaves (Dong et al., 2019). The calculation formula is as follows:


$$SII\;=\;(A_{T}-B_{T})/(A_{CK}-B_{CK})$$

In the formula, SII is the salt injury index of physiological and phenotypic traits, A_T_ is the day after NaCl treatment phenotypic value, B_T_ is the day before NaCl treatment (0 days) phenotypic value, A_CK_ is the day after control phenotypic value, and B_CK_ is the day before the control (0 days) phenotypic value.

### Total RNA extraction, library preparation and assembly, and analysis

For transcriptomic analysis, complete seedling tissue samples were collected at 0 DAT, 3 DAT, 6 DAT, and 9 DAT. At each time point, seedlings from two maize inbred lines were carefully harvested, and the roots were immediately rinsed with distilled water to remove soil particles. To prevent RNA degradation, samples were wrapped in tin foil, rapidly frozen in liquid nitrogen, and then stored in an ultra-low temperature freezer at −80°C for long-term preservation. At the onset of salt stress, plants undergo a series of physiological and molecular responses. However, these changes are not immediately detectable, and previous studies have not observed significant differences between the control and salt-treated groups at this time point. Therefore, only samples from the CK group were collected at 0 DAT, with each sample comprising three biological replicates.

Total RNA was extracted from 21 tolerant and 21 sensitive maize seedling tissue samples using TRIzol^®^ Reagent (Invitrogen, Carlsbad, CA, USA), following the manufacturer’s instructions. RNA quality was assessed using the 5300 Bioanalyzer (Agilent, Santa Clara, CA, USA) and quantified using the ND-2000 spectrophotometer (NanoDrop Technologies, Wilmington, DE, USA). Only high-quality RNA samples (OD260/280 = 1.8–2.2, OD260/230 ≥ 2.0, RQN ≥ 6.5, 28S:18S ≥ 1.0, and ≥1 μg) were used for sequencing library construction. RNA sequencing (RNA-seq) libraries were prepared using the Illumina^®^ Stranded mRNA Prep, Ligation Kit (San Diego, CA, USA), where mRNA was isolated via poly(A) selection with oligo(dT) beads and fragmented. The fragmented RNA was reverse-transcribed into double-stranded cDNA, which was then end-repaired, phosphorylated, and ligated with adapters. Size selection was performed for cDNA fragments of 300–400 bp using magnetic beads, followed by PCR amplification for 10–15 cycles. Libraries were quantified using Qubit 4.0, and sequencing was performed on the NovaSeq 6000 platform (Illumina, USA) by Shanghai Majorbio Bio-pharm Technology Co., Ltd., Shanghai, China. The raw paired-end reads were trimmed and quality-controlled by fastp (Chen et al., 2018) with default parameters. Then, clean reads were separately aligned to Zm-Mo17-REFERENCE-CAU-2.0.fa (https://maizegdb.org) reference genome, the expression levels were quantified, and the reads were converted into fragments per kilobase of transcript sequence per million base pairs sequenced (FPKM) (Li and Dewey, 2011) with orientation mode using the HISAT2 (Li et al., 2009; Kim et al., 2015) software. The mapped reads of each sample were assembled by StringTie (Pertea et al., 2015) in a reference-based approach. The mapping results returned a read count for each transcript. RSEM (Li and Dewey, 2011) was used to qualify gene abundances. Genes with an FPKM value ≥1 were considered to be expressed to ensure consistent and meaningful analyses; FPKM values were normalized and analyzed. FPKM values, which were already adjusted for sequencing depth and gene length, were further normalized using min–max scaling to a 0–1 range. This additional normalization was performed using the formula:


$$\text{Normalized value}=\frac{\text{FPKM value}-\text{min }(\text{FPKM})}{\max(\text{FPKM})-\text{min }(\text{FPKM})}$$

Normalized expression values were used to assess gene expression levels and identify patterns using hierarchical clustering and K-means clustering methods in the MeV (V4.9) software and gene expression heatmap using the pheatmap R package.

### Differentially expressed genes and protein–protein interaction

The DESeq2 (1.26.0) (Wang et al., 2009) R package was then used to normalize the read counts, and a differential expression analysis was performed based on the negative binomial distribution for its estimation model (Anders and Huber, 2010). To control the false discovery rate (FDR), Benjamini and Hochberg’s method (Yoav Benjamini, 1995) was used to calculate adjusted *p*-values, and the significance threshold for the adjusted *p*-values was set to α = 0.05. Further screening of DEGs from expressed genes requires genes considered significantly differentially expressed to meet both |log2 (fold change)| ≥ 1.5 and *p*-value ≤ 0.01.

Protein–protein interactions (PPIs) were analyzed by the STRING database (https://string-db.org/) (Szklarczyk et al., 2015) using the proteins by sequences as input, and a confidence score >0.4 and *p* < 0.05 were set as the cutoff criteria.

### Identification and functional annotation of differentially expressed genes

To gain deeper insights into the functional roles of DEGs in maize seedlings under salt stress at various time intervals, Gene Ontology (GO) enrichment and Kyoto Encyclopedia of Genes and Genomes (KEGG) pathway enrichment analyses were performed. The KEGG database by the online tool KEGG Orthology-Based Annotation System (KOBAS 3.0, http://KOBAS.cbi.pku.edu.cn) (Bu et al., 2021) was utilized for KEGG pathway enrichment analysis. The criterion for substantially enriched KEGG pathways was a *p*-value < 0.05. GO classification was conducted to identify significantly enriched terms (*p*-value < 0.05) for biological process (BP), molecular process (MP), and cellular component (CC). These were further identified using the PlantRegMap online tool (http://plantregmap.cbi.pku.edu.cn/go_result.php, accessed on 27 May 2022). Furthermore, REVIGO (http://revigo.irb.hr/) (Supek et al., 2011) was used to visualize the top significantly enriched GO terms (*p*-value < 0.001).

### Weighted gene co-expression network analysis

Co-expression networks were constructed via the WGCNA package in R (Langfelder and Horvath, 2008) from the RNA-seq data of SPL02 and Mo17 co-expression DEGs in the salt treatment group. FPKM values were normalized, and an adjacency matrix was constructed. The soft-thresholding power (β) was selected using the pickSoftThreshold function to achieve a scale-free topology (Zhang et al., 2005). A power of 12 was chosen. An adjacency matrix was constructed and transformed into a topological overlap matrix (TOM) to measure network interconnectedness (Yip and Horvath, 2007). Genes were clustered based on TOM dissimilarity, and modules were detected using dynamic tree cutting. Modules with similar expression profiles were merged based on the hierarchical clustering of module eigengenes. Module eigengenes (MEs) were calculated as the first principal component of each module. The relationship between MEs and external traits was evaluated using Pearson’s correlations, with significant modules further analyzed for biological significance. Gene significance (GS) was defined as the absolute value of the correlation between the gene expression profile and the trait of interest. Module membership (MM) was calculated as the correlation between the gene expression profile and the module eigengene. Scatter plots of MM versus GS were generated to identify hub genes within significant modules. Module-specific gene networks generated by WGCNA were visualized using the cytoHubba plugin in Cytoscape 3.9.1 (Shannon et al., 2003).

### Real-time quantitative PCR

Real-time quantitative PCR (qRT-PCR) was conducted to validate the expression patterns of the hub genes. Total RNA was isolated utilizing TRIzol^®^ reagent (Invitrogen), and PrimeScript™ RT reagent kit with gDNA Eraser kit (Takara, Mountain View, CA, USA) was used to eliminate genomic DNA contamination and synthesize cDNA. Quantitative PCR was conducted using the TB Green^®^ Premix Ex Taq™ II Kit (Takara) on an ABI 7500 Real-Time PCR Detection System (Applied Biosystems, Foster City, CA, USA). The thermal cycling conditions were as follows: pre-denaturation at 95°C for 30 s, followed by 40 cycles of 95°C for 5 s and 60°C for 34 s. The melting curve program included 95°C for 15 s, 60°C for 1 min, and 95°C for 15 s. The relative transcription levels of selected genes were calculated using the 2^−ΔΔCt^ method (Livak and Schmittgen, 2001). qRT-PCR primers are listed in **Supplementary Table S3**.

## Results

### Morphological and physiological analyses of SPL02 and Mo17 seedlings’ response to salt stress

**Figure 1:**
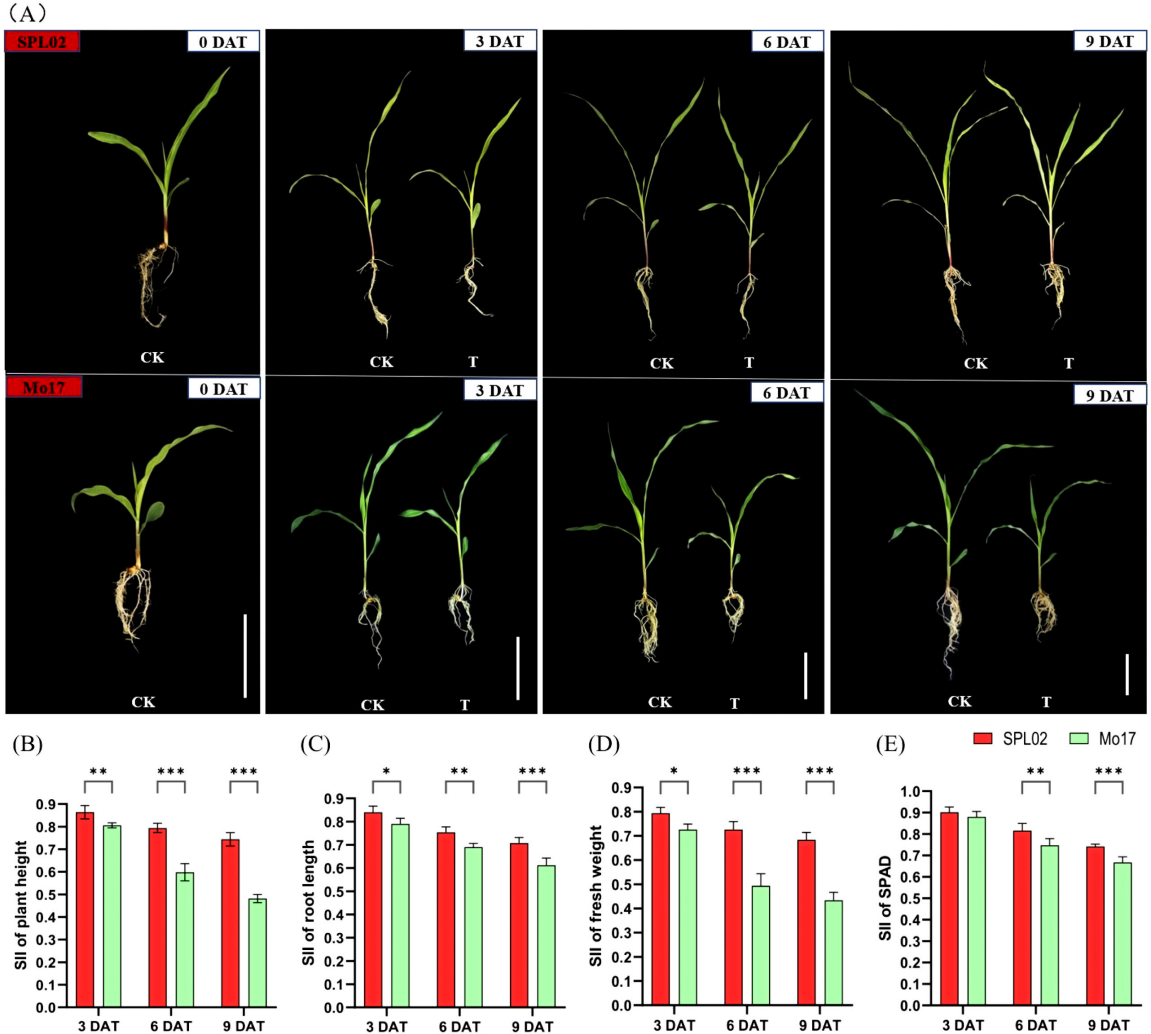
Phenotypic and physiological responses to salt stress in two maize inbred lines with NaCl treatment and control at different times. **(A)** Phenotypic comparison of seedling stage plants between maize inbred line SPL02 and maize inbred line Mo17. DAT represents the days after salt treatment; CK represents the control conditions; T represents the salt treatment conditions; bar = 7 cm. **(B)** SII of plant height at 3 DAT, 6 DAT, and 9 DAT. **(C)** SII of root length at 3 DAT, 6 DAT, and 9 DAT. **(D)** SII of fresh weight at 3 DAT, 6 DAT, and 9 DAT. **(E)** SII of chlorophyll content (SPAD) at 3 DAT, 6 DAT, and 9 DAT. Note: Values are mean ± SD for each measurement. *, **, and *** indicate significance level at *p* < 0.05, *p* < 0.01, and *p* < 0.001, respectively.

We studied the morphological and physiological responses of two maize inbred lines, SPL02 and Mo17; no visible phenotypic differences were observed between the two lines in the CK group. However, by 6 DAT and 9 DAT, Mo17 seedlings exhibited higher susceptibility and slower growth compared to SPL02, which displayed only minor phenotypic stress (**Figure 1A**).

This observation revealed that SPL02 possesses a better salt tolerance than Mo17. To understand the phenotypic and physiological responses to NaCl treatment, we analyzed the salt injury index (SII) across various traits. The SII of plant height decreased significantly with increased salt treatment duration in both SPL02 and Mo17 (**Figure 1B**). Specifically, the SII of plant height in SPL02 decreased by 86.5%, 79.5%, and 74.3% at 3 DAT, 6 DAT, and 9 DAT, respectively, while in Mo17, it decreased by 80.6%, 59.7%, and 48.0% at the same time points. Plant height exhibited a notable decline at 6 DAT and 9 DAT in Mo17. Similarly, the SII of root length showed a marked decline with prolonged salt treatment (**Figure 1C**). Over time, the SII of root length continued to decrease, with Mo17 displaying a more pronounced reduction. The SII of fresh weight also dropped sharply with increased salt concentration (**Figure 1D**). The SII of fresh weight in SPL02 declined by 79.6%, 72.4%, and 68.4% at 3 DAT, 6 DAT, and 9 DAT, respectively, while in Mo17, it decreased by 72.4%, 49.4%, and 43.3% at the same time points. Fresh weight showed a significant decline at 6 DAT and 9 DAT in Mo17. Lastly, the SII of SPAD decreased with salt treatment (**Figure 1E**). With extended salt treatment, the SII of SPAD declined, with Mo17 showing a more significant decrease. These results indicate that SPL02 maintains better overall performance under salt stress compared to Mo17, demonstrating its superior salt tolerance.

### Illumina paired-end sequencing and assembly of maize transcriptomes

We conducted an experiment involving the salt-tolerant inbred line SPL02 and the salt-sensitive inbred line Mo17 under varying durations of the CK and T conditions to elucidate the regulatory mechanisms underlying the response of maize seedlings to salt stress. We collected samples at 0 DAT, 3 DAT, 6 DAT, and 9 DAT, with each time point including three biological replicates for RNA sequencing. In total, we obtained 316.89 Gb of raw data. The effective data volume per sample ranged from 6.45 to 8.42 Gb, with Q30 base distribution values between 94.95% and 95.55% and an average GC content ranging from 48.98% to 55.35% (**Supplementary Table S1**).

The clean reads were mapped to the Mo17 maize reference genome (Zm-Mo17-REFERENCE-CAU-2.0.fa), with 42,580 genes identified. Pearson’s correlation coefficients revealed an average correlation of 0.92 among the three biological replicates within each sample across the two maize lines, indicating strong consistency (**Supplementary Figure S1**). Furthermore, principal component analysis (PCA) of the expressed genes revealed that the first two principal components (PCs) accounted for 79.47% of the total variance, with PC1 explaining 58.07% and clearly distinguishing between different salt treatment durations, while PC2 accounted for 21.4% and differentiated between SPL02 and Mo17 (**Supplementary Figure S2**). These results validate the reliability and robustness of the transcriptomic data, providing a solid foundation for subsequent analyses.

### Expression trends of genes at different time points

**Figure 2:**
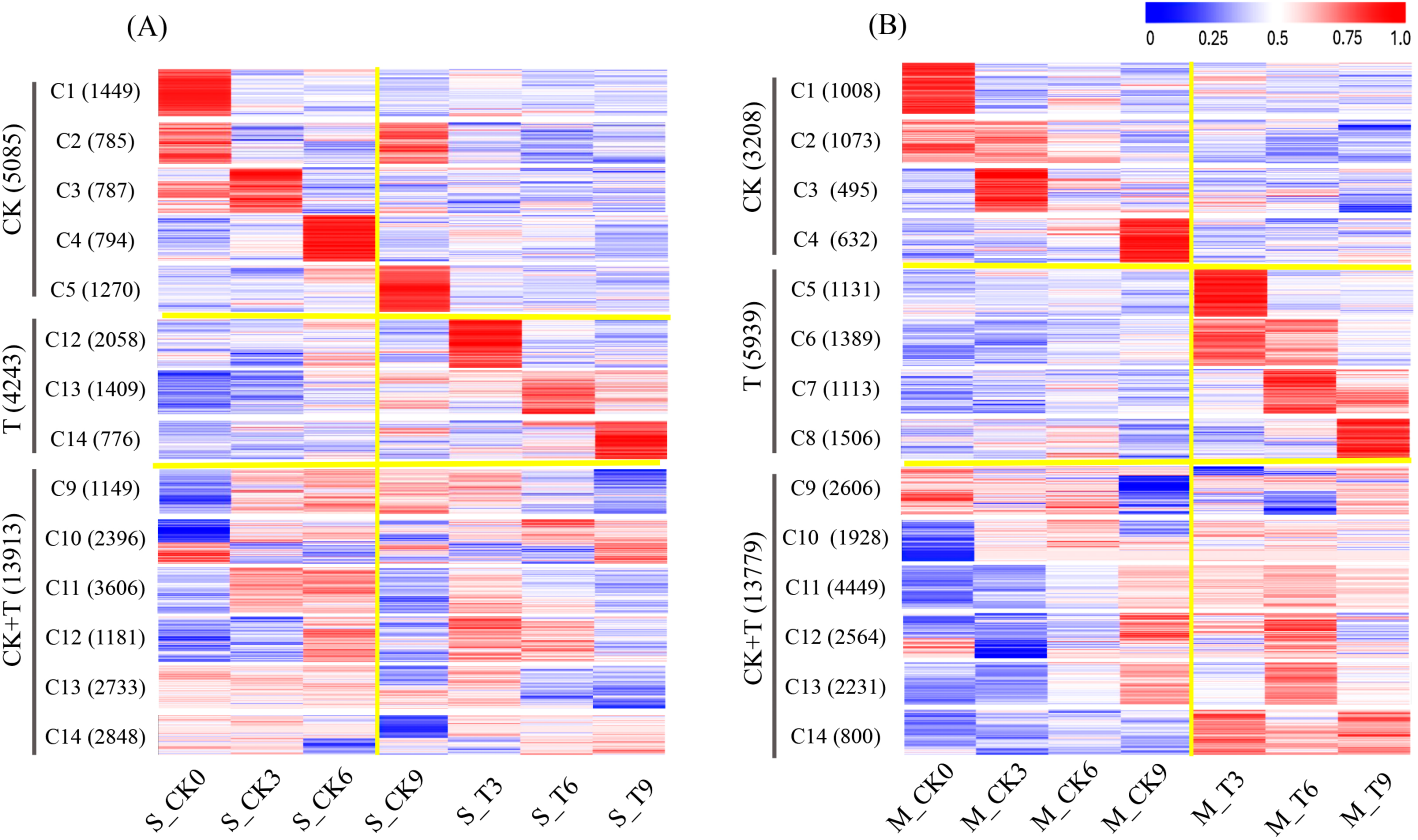
Clustered heatmap of gene expression patterns in SPL02 and Mo17. The FPKM value normalized by the maximum value of all FPKM values of expressed genes across all seven phases is shown for each gene. The expressed genes are clustered into 14 clusters. The sequence of clusters is shown on the left. The number of genes in each cluster is shown on the right. **(A)** Clustered heatmap of gene expression under CK, T, and CK+T expression categories in SPL02. **(B)** Clustered heatmap of gene expression under CK, T, and CK+T expression categories in Mo17.

Among the 42,580 protein-coding genes identified, 25,383 genes with an FPKM value of ≥1 were considered expressed genes. The actively expressed genes were subsequently grouped into 14 clusters within each of the two maize inbred lines, with each cluster representing distinct expression categories across the experimental conditions: CK, T, and CK+T (shared response). Genes highly expressed in the CK category support normal growth and development. Genes highly expressed in the T category primarily mediate adaptation to salt stress. Genes within the CK+T category play dual roles in growth and stress response, highlighting their functional versatility. A total of 23,241 expressed genes were detected in SPL02 (**Figure 2A**). Specifically, 5,085 highly expressed genes belonged to CK, 4,243 highly expressed genes belonged to T, and 13,913 highly expressed genes belonged to CK+T. It was observed that the number of expressed genes in SPL02 declined as the duration of salt treatment increased.

In contrast, a total of 22,926 expressed genes were detected in Mo17 (**Figure 2B**). The proportions were as follows: 3,028 expressed genes with high expression under CK, 5,939 expressed genes with high expression under T, and 13,779 expressed genes with high expression under CK+T. Notably, Mo17 had a higher number of expressed genes under salt treatment compared to SPL02, and this number increased with the duration of exposure, particularly evident at 3 DAT, 6 DAT, and 9 DAT. These findings underscore the dynamic nature of gene expression in response to salt stress, revealing distinct responses between SPL02 and Mo17. The differential expression patterns observed over time offer valuable insights into the mechanisms of salt tolerance in maize.

### Dynamic expression of differentially expressed genes and protein–protein interaction analysis

DEGs were identified based on their FPKM values, with criteria of |log2 FC| ≥ 1.5 and a *p*-value ≤ 0.01. A total of 8,971 DEGs were identified between the two maize inbred lines (**Table 1**). Specifically, 7,111 genes were uniquely expressed in SPL02, representing 79.26% of the DEGs, while 4,791 genes were uniquely expressed in Mo17, accounting for 53.40% of the DEGs. Additionally, 2,931 DEGs were commonly expressed in both inbred lines (**Supplementary Figure S3**).

**Table 1:** Comparison of DEGs between two inbred lines at different time points.

Items	Mo17			SPL02		
	Total	Up	Down	Total	Up	Down
T3_vs_CK0	1,676	826	850	3,166	1,698	1,468
T3_vs_CK3	482	318	164	1,250	781	469
T6_vs_CK0	2,944	724	2,220	3,363	1,814	1,549
T6_vs_CK6	1,090	221	869	1,632	825	807
T9_vs_CK0	2,250	758	1,492	2,708	1,280	1,428
T9_vs_CK9	1,214	772	442	1,764	470	1,294

DEGs, differentially expressed genes.

**Figure 3:**
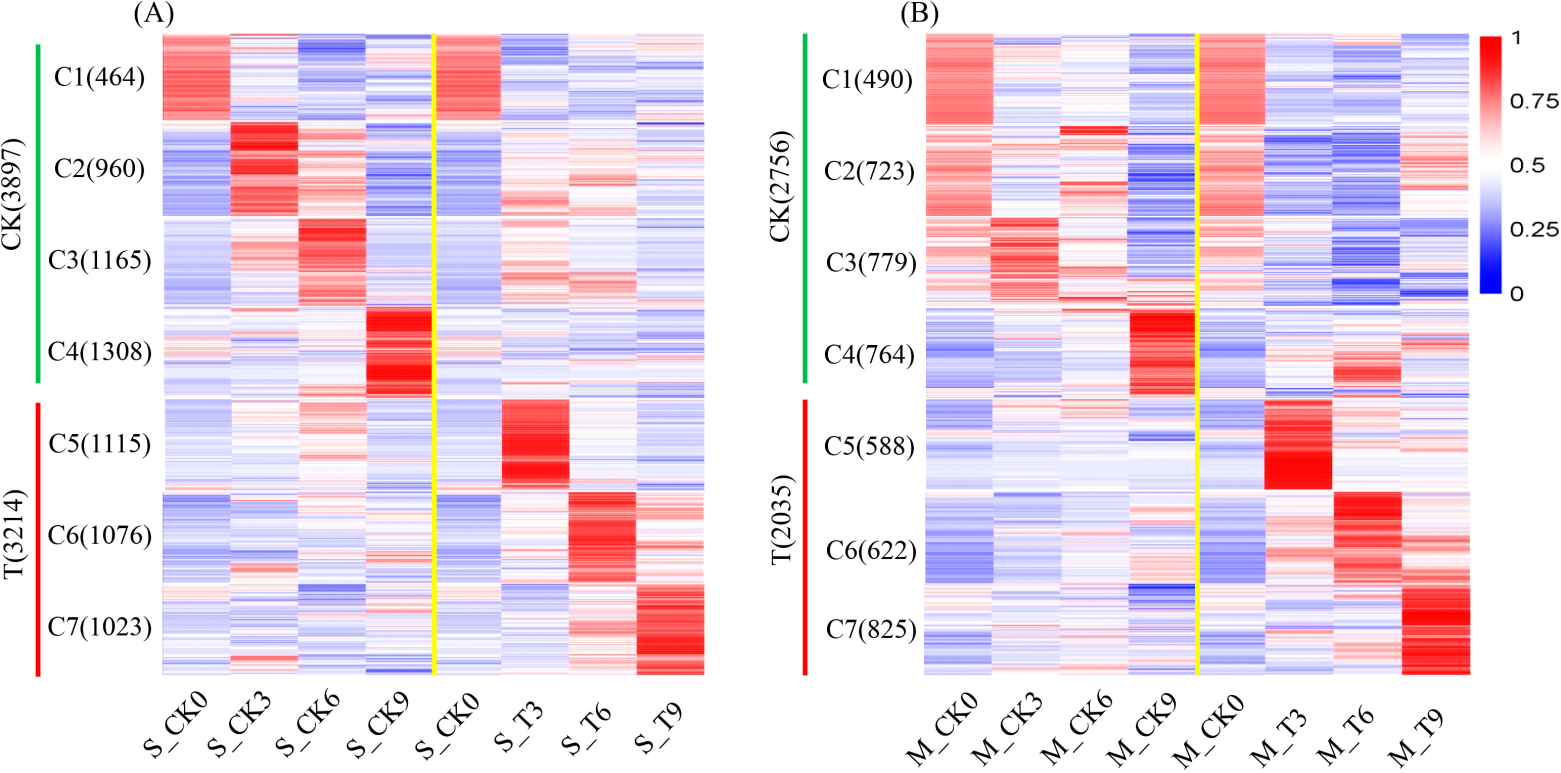
Clustered heatmap of DEG expression patterns in SPL02 and Mo17. The FPKM value normalized by the maximum value of all FPKM values of expressed genes across all seven phases is shown for each gene. The expressed genes are clustered into seven clusters. The clustering sequence and the number of genes contained in each cluster are shown on the left. **(A)** Clustered heatmap of DEGs under CK and T categories in SPL02. **(B)** Clustered heatmap of DEGs under CK and T categories in Mo17.

To comprehend the expression trends of DEGs in two inbred lines at various time points following salt treatment, DEGs were partitioned into seven clusters. In DEG expression clusters of SPL02 (**Figure 3A**), 3,897 genes showed heightened expression in the CK group, constituting 55% of DEGs; 3,214 genes showed heightened expression in the T group, accounting for 45% of DEGs. In DEG expression clusters of Mo17 (**Figure 3B**), 2,756 genes exhibited high expression levels in the CK group, constituting 57.52% of the DEGs; 2,035 genes showed heightened expression in the T group, accounting for 42.47% of DEGs. In addition, as the salt treatment time prolonged, the number of genes expressed increased in Mo17, while it decreased in SPL02.

**Figure 4:**
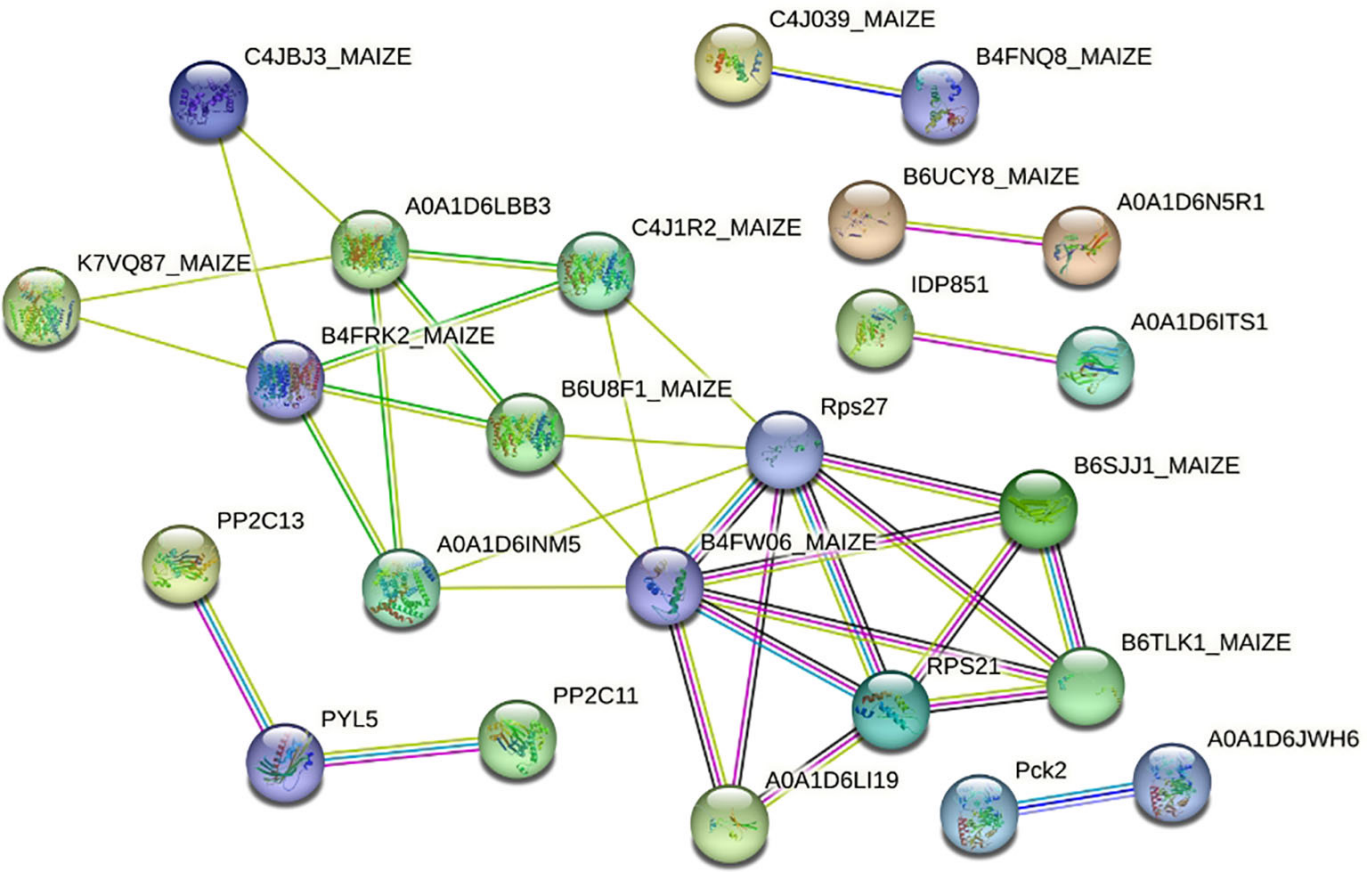
The protein interaction network of two maize inbred lines’ DEGs related to salt stress. DEGs, differentially expressed genes.

Through a review of studies related to salt tolerance in maize (Sun et al., 2018; Xie et al., 2019; Zhao et al., 2019; Sandhu et al., 2020; Kong et al., 2021; Li et al., 2021; Zhang et al., 2021b, 2023; Cao et al., 2022), 89 genes were identified as being co-expressed with SPL02 and Mo17 DEGs. Further protein–protein interaction analysis was conducted on these 89 genes to investigate the signal transduction of salt stress in maize seedlings. This analysis revealed 25 genes organized into six distinct protein interaction groups (**Figure 4**; **Supplementary Table S2**). The first group includes proteins related to ammonium transporters, potassium outward rectifying channels, sodium/hydrogen exchangers, and ribosomal proteins. The second group consists of proteins associated with 2C-type protein phosphatase and pyrabactin resistance-like proteins. The third group contains proteins linked to phosphoenolpyruvate carboxykinase. The fourth group features proteins related to putative calcium-binding proteins. The fifth group includes proteins within the RNA-binding family. The sixth group encompasses proteins involved in salt stress response and ethylene signaling.

### Functional enrichment analysis of differentially expressed genes

To reveal the relationship between DEGs and salt stress in maize, KEGG and GO enrichment analyses were conducted for each cluster to elucidate the functional roles of these DEGs. Pathways with a *p*-value < 0.05 and containing more than three genes were considered significant. From these, the top 20 KEGG pathways and the top 30 GO terms were selected for detailed analysis. The GO terms and KEGG pathways highlighted key roles in maize adaptation to salt stress, which are essential for maintaining cellular homeostasis and regulating energy processes.

**Figure 5:**
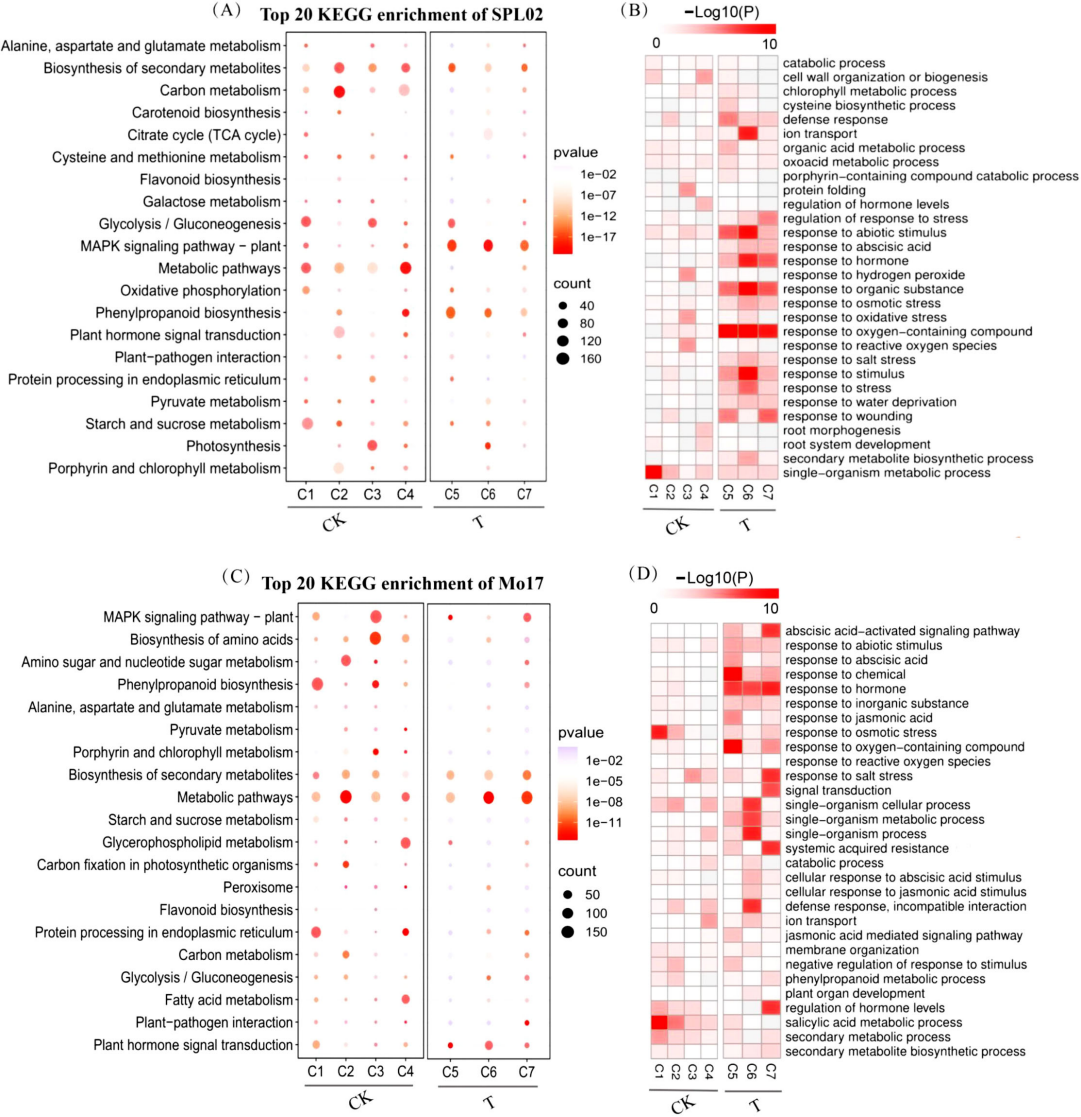
KEGG pathway and GO term enrichment analyses of Mo17 and SPL02 exposed to different treatment stages. **(A)** KEGG pathway enrichment of SPL02. **(B)** GO enrichment of SPL02 in seven clusters. The *p*-value indicates the significance of GO terms. **(C)** KEGG pathway enrichment of Mo17. **(D)** GO enrichment of Mo17 in seven clusters. The *p*-value indicates the significance of GO terms. KEGG, Kyoto Encyclopedia of Genes and Genomes; GO, Gene Ontology.

In SPL02, KEGG enrichment results revealed that in the CK group, significant pathways included those involved in the biosynthesis of secondary metabolites, carbon metabolism, glycolysis/gluconeogenesis, metabolic pathways, and cysteine and methionine metabolism. In contrast, significant enrichment was observed in pathways related to secondary metabolites in the T group, the MAPK signaling pathway in plants, and phenylpropanoid biosynthesis (**Figure 5A**). GO enrichment analysis showed that pathways related to signal transduction and organic metabolic processes were significantly enriched in the CK group. Following salt treatment, pathways associated with responses to abiotic stimuli, hormone signaling, organic substances, and oxygen-containing compounds were notably enriched (**Figure 5B**).

Similarly, in Mo17, KEGG pathways were analyzed for enrichment (**Figure 5C**). The analysis revealed significant enrichment in pathways related to the biosynthesis of secondary metabolites, biosynthesis of amino acids, the MAPK signaling pathway in plants, and general metabolic pathways in the CK group. Significant enrichment was observed in pathways associated with the biosynthesis of secondary metabolites and metabolic pathways in the T group. GO enrichment analysis indicated that, in the CK group, salicylic acid metabolic processes and responses to osmotic stress were significantly enriched. Significant enrichment was observed in pathways related to the abscisic acid-activated signaling pathway, hormone responses, and salt stress responses in the T group (**Figure 5D**).

These comprehensive analyses offer valuable insights into the transcriptional dynamics and molecular mechanisms underlying salt stress responses in SPL02 and Mo17. This information is crucial for understanding the genetic and biochemical pathways that contribute to salt tolerance in maize, offering potential targets for enhancing salt resistance through molecular breeding strategies.

**Figure 6:**
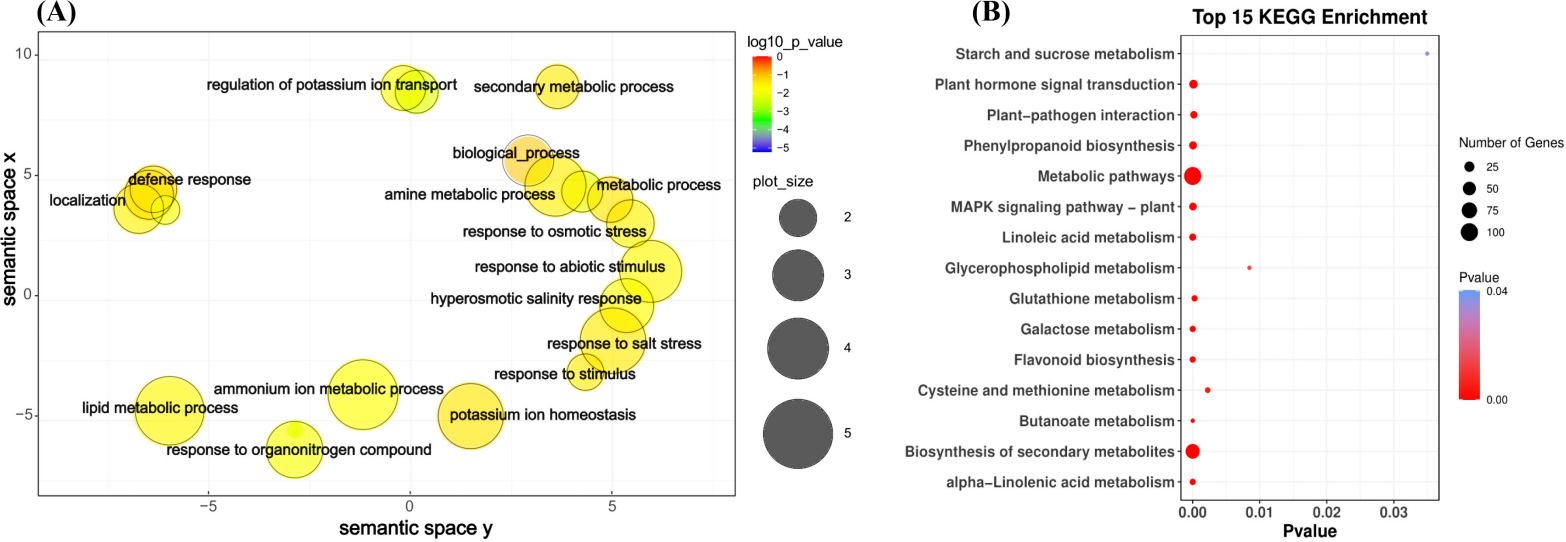
GO term and KEGG pathway enrichment assigned to the salt treatment (T) for 597 differential genes between SPL02 and Mo17. **(A)** GO categories for biological process. **(B)** Top 15 KEGG enrichment pathways. KEGG, Kyoto Encyclopedia of Genes and Genomes; GO, Gene Ontology.

Among the seven gene expression clusters across two maize inbred lines, 597 genes were identified to be highly expressed in the T group in both lines (**Supplementary Figure S4**). To further investigate the functional roles of these genes, a GO enrichment analysis was conducted. The top GO terms with high significance (*p* < 0.001) were further analyzed using REVIGO to provide a more concise visualization. Key biological processes related to salt stress identified through this analysis included response to abiotic stimulus, response to osmotic stress, response to salt stress, and hyperosmotic salinity response (**Figure 6A**). In terms of molecular functions, DNA-binding transcription factor activity and oxidoreductase activity were prominently identified as significant (**Supplementary Figure S5**). These results highlight the critical biological processes and molecular functions associated with maize’s response to salt stress, providing a clearer understanding of the underlying mechanisms and pathways involved in salt tolerance.

Similarly, KEGG enrichment analyses were conducted for 597 genes. The top 15 KEGG pathways were selected for detailed analysis (**Figure 6B**). The KEGG pathway enrichment analyses revealed that the metabolic pathway category has the most associated genes. This suggests that a broad range of genes are involved in general metabolic processes, which are critical for maintaining cellular function and energy balance under salt stress. In addition to metabolic pathways, other notable pathways, such as biosynthesis of secondary metabolites, plant hormone signal transduction, phenylpropanoid biosynthesis, and MAPK signaling pathway - plant, also show significant enrichment. These pathways are essential for regulating the plant’s stress response through signaling mechanisms.

### Identification of key modules possessing candidate genes via WGCNA

To explore the relationship between DEGs responding to alkaline salt stress and physiological and phenotypic indicators across the two maize inbred lines, we conducted a WGCNA. We focused on 597 candidate genes identified in the T group in both inbred lines for this analysis.

**Figure 7:**
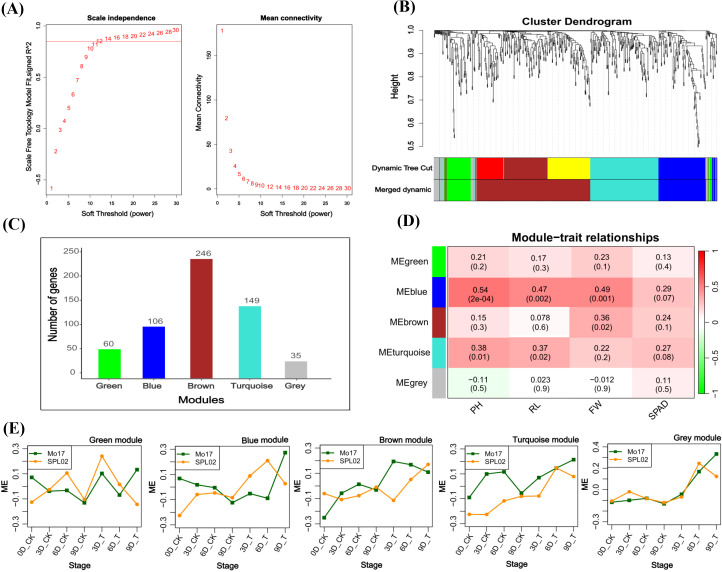
**(A)** Plots of the soft threshold vs. scale independence and mean connectivity. **(B)** Module clustering; different colors represent different modules. **(C)** Number of genes in each module. **(D)** Module trait relationship; each row corresponds to a module, while each column corresponds to the salt tolerance physiological and phenotypic indicators. The left panel shows the modules, while the right panel shows positive (red, 1) and negative (green, −1) correlations. **(E)** Module eigengenes’ expression patterns of the modules at different salt stress developmental stages in two maize inbred lines.

The initial step involved selecting an appropriate power value (β) to ensure optimal network connectivity. By testing various β values, we found that a β of 12 achieved an R^2^ value greater than 0.8 and a sufficiently high mean connectivity, making it the final choice for our analysis (**Figure 7A**). We then categorized these genes into five distinct modules based on their expression patterns: green (60 genes), blue (106 genes), brown (246 genes), turquoise (149 genes), and gray (35 genes) (**Figures 7B, C**). To assess the relevance of these modules to salt stress, we performed Pearson’s correlation analysis. The blue module showed significant positive correlations with plant height (PH) (R = 0.54, p = 2e^−04^), root length (RL) (R = 0.47, p = 0.002), and fresh weight (FW) (R = 0.49, p = 0.001) (**Figure 7D**). Notably, genes in the blue module were more highly expressed during salt treatment in Mo17 compared to SPL02, reflecting the differing salt stress responses observed in the two inbred lines (**Figure 7E**). These results underscore the importance of the blue module genes in mediating salt tolerance-related traits. Therefore, we decided to further investigate the genes within the blue module to gain deeper insights into their roles in maize’s response to salt stress.

**Figure 8:**
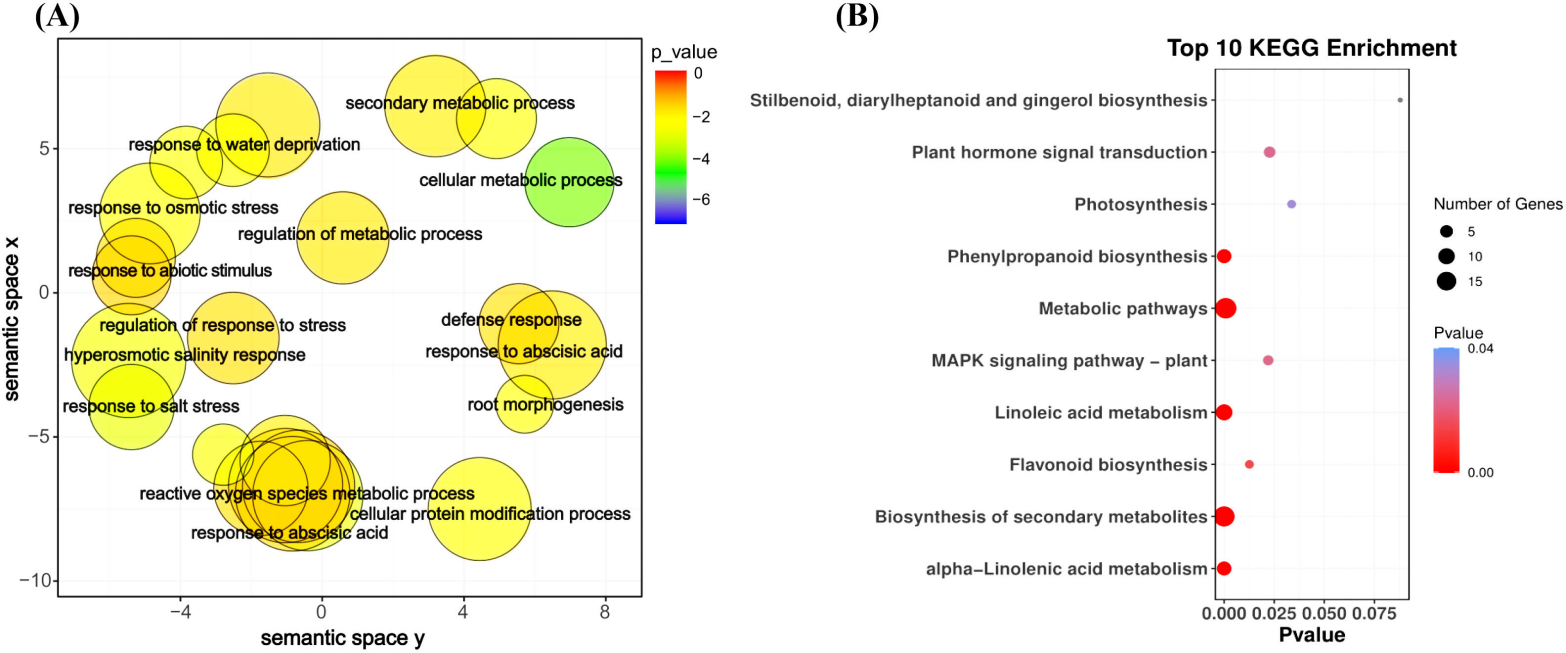
GO term and KEGG pathway enrichment for blue module. **(A)** GO categories for biological process, blue module. **(B)** KEGG enrichment pathways, blue module. KEGG, Kyoto Encyclopedia of Genes and Genomes; GO, Gene Ontology.

**Figure 9:**
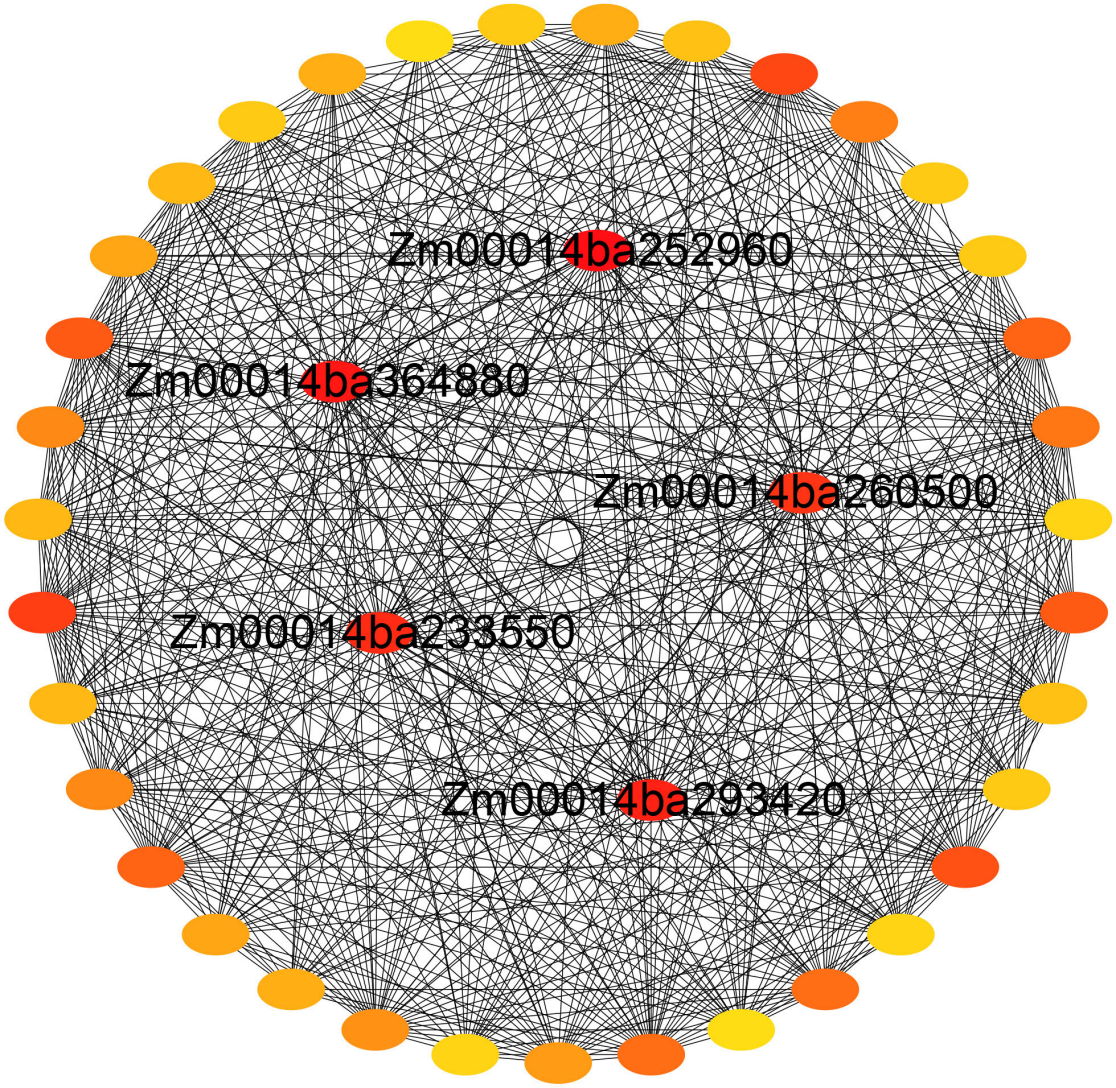
Gene network for the blue module.

**Figure 10:**
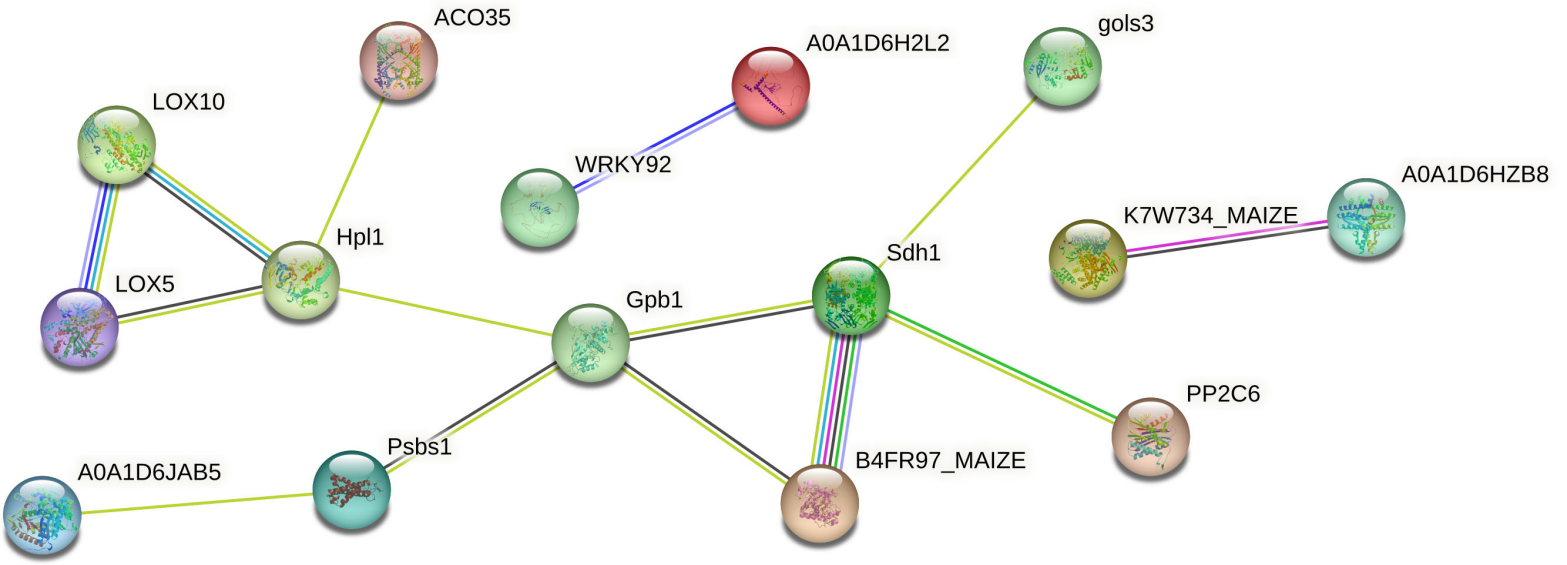
The protein interaction network of top 10 KEGG pathways in blue module and hub genes. KEGG, Kyoto Encyclopedia of Genes and Genomes.

The GO enrichment analysis of the blue module revealed several significant findings. Notably, the biological processes associated with this module include genes related to key functions such as defense response (GO:0006952), response to osmotic stress (GO:0006970), response to water deprivation (GO:0009414), response to salt stress (GO:0009651), response to abscisic acid (GO:0009737), and hyperosmotic salinity response (GO:0042538) (**Figure 8A**). In terms of molecular functions, the genes in the blue module are linked to glutamate decarboxylase activity (GO:0004351) and oxidoreductase activity (GO:0016491) (**Supplementary Figure S6**). The top 10 KEGG pathway enrichment analysis reveals that the metabolic pathways, biosynthesis of secondary metabolites, linoleic acid metabolism, phenylpropanoid biosynthesis, plant hormone signal transduction, and MAPK signaling pathway category have the most associated genes (**Figure 8B**). Recent findings suggest that genes within the blue module are potentially critical for the response of maize seedlings to salt stress and may play significant roles in salt tolerance. To identify hub genes within this module, we visualized gene networks using Cytoscape 3.9.1. This analysis identified five major hub genes: phosphate import ATP-binding protein (PstB1), glycosyltransferase, WRKY transcription factor WRKY71, lipoxygenase 2.3 (chloroplastic), and peroxisomal membrane protein 11-5 (**Figure 9**; **Table 2**; **Supplementary Table S4**). To explore the relationship between the hub genes and KEGG pathways, we analyzed the interaction of the five hub genes with the genes with enrichment in the top 10 KEGG pathways in the blue module. We performed a protein–protein interaction analysis and organized the results into three distinct protein interaction groups. The results indicate that *Zm00014ba260500* (protein annotation: A0A1D6H2L2, Putative WRKY transcription factor 40) interacts with *Zm00014ba373230* (protein annotation: WRKY92, Putative WRKY DNA-binding domain superfamily protein isoform 1), which is enriched in the MAPK signaling pathway - plant. Additionally, *Zm00014ba233550* (protein annotation: LOX10, Lipoxygenase) interacts with 10 proteins, and these gene interactions are significantly enriched in the KEGG pathways of metabolic pathways, linoleic acid metabolism, alpha-linolenic acid metabolism, biosynthesis of secondary metabolites, MAPK signaling pathway - plant, and photosynthesis (**Figure 10**; **Supplementary Table S5**). These hub genes are likely to influence salt tolerance either directly or indirectly, highlighting their crucial roles in the mechanisms of salt tolerance in maize.

**Table 2:** Five hub genes identified in the blue module of WGCNA.

Gene-ID	Weight	Annotation
Zm00014ba252960	0.26329	Phosphate import ATP-binding protein PstB1 [*Zea mays*]
Zm00014ba364880	0.17309	Glycosyltransferase [*Z. mays*]
Zm00014ba260500 Zm00014ba233550	0.15816 0.15002	WRKY transcription factor WRKY71 [*Z. mays*] Lipoxygenase 2.3, chloroplastic [*Z. mays*]
Zm00014ba293420	0.11994	Peroxisomal membrane protein 11-5 [*Z. mays*]

WGCNA, weighted gene co-expression network analysis.

**Figure 11:**
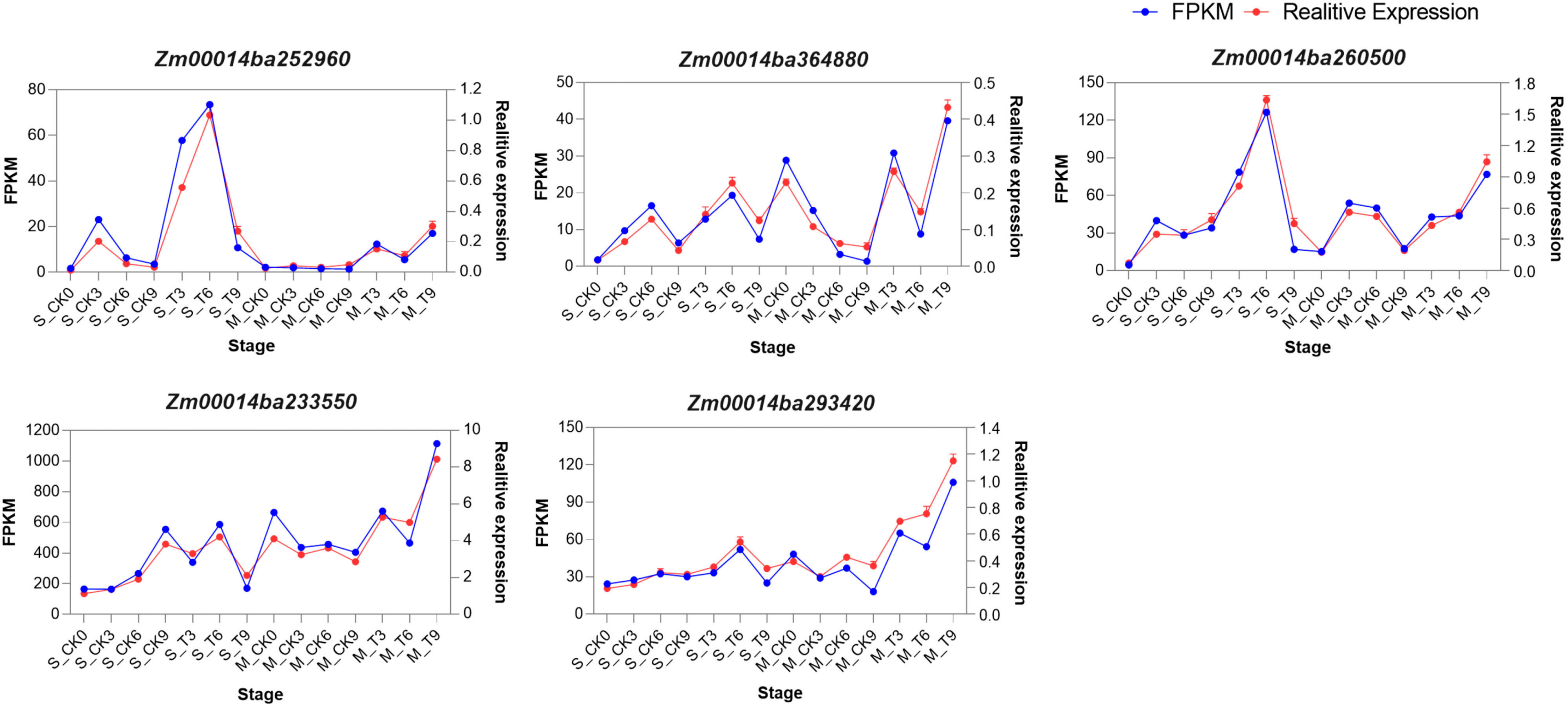
Expression pattern analysis of five hub genes by RNA-seq and qRT-PCR. The y-axis shows the mRNA levels. The scale on the left indicates gene expression level based on RNA-seq. The scale on the right indicates relative expression based on qRT-PCR. The x-axis indicates the phases of control and salt treatment. The blue lines correspond to RNA-seq, while the red lines correspond to qRT-PCR.

To assess the reliability of gene expression profiles and determine the consistency of WGCNA results with experimental data, we validated five hub genes using qRT-PCR under both CK and T conditions. The results of qRT-PCR exhibited similar expression patterns as the RNA-seq results (**Figure 11**). Based on these results, we confirmed that RNA-seq was highly consistent with qRT-PCR.

## Discussion

### Morphological and physiological responses in two inbred lines under salt stress

Salt stress is a well-documented abiotic factor that disrupts plant growth, affecting physiological and biochemical activities, including chlorophyll content and ion homeostasis (Affenzeller et al., 2009; Shahzad et al., 2019). In this study, we assessed the salt tolerance in SPL02 and Mo17 under different salt treatment durations. SPL02 maintained robust growth across 3 DAT, 6 DAT, and 9 DAT, while Mo17 exhibited reduced growth, particularly after extended exposure. These findings align with previous studies, demonstrating that salt-sensitive lines experience greater growth inhibition and physiological disruptions under salt stress (Chen et al., 2019; Zhang et al., 2021b). By analyzing phenotypic traits across treatment periods, we observed that SPL02 consistently exhibited higher indices, underscoring its enhanced salt tolerance. These metrics reflect SPL02’s ability to maintain cellular integrity and mitigate ion leakage, which is a response often used to assess membrane stability under salt stress (Chen et al., 2013). It is worth noting that under T conditions, SII of plant height and SII of fresh weight were significantly lower than other indicators. These findings align with previous studies (Kumar et al., 2021), indicating that the accumulation of salt will cause dehydration of plant cells, resulting in a decrease in cell expansion capacity, thereby affecting fresh weight and inhibiting plant growth (Sadiq et al., 2024; Xu et al., 2024). This study supports the observation that morphological and physiological differences play critical roles in the contrasting salt stress responses between salt-tolerant and salt-sensitive maize.

### Dynamic change of expression genes and DEGs

Plants adjust gene expression dynamically, transitioning from initial responses to long-term adaptations (Waadt et al., 2022). Salt tolerance in plants is a quantitative trait governed by the interplay of multiple genes, contributing to the complexity of salt tolerance mechanisms (Foolad and Jones, 1993). Gene expression revealed more expressed genes in SPL02 than in Mo17, suggesting that SPL02’s gene regulation in response to salt stress is more intricate. These dynamic gene expression changes across 14 gene clusters in both SPL02 and Mo17 suggest distinct molecular mechanisms and stress-response strategies tailored to salt tolerance in each inbred line. Salt stress initiates an immediate osmotic response due to high NaCl concentration, followed by ionic stress as Na^+^ levels in the cytoplasm approach toxicity (Wang et al., 2020). Notably, at 3 DAT, SPL02 and Mo17 displayed heightened expression of many expressed genes, indicating the activation of initial stress responses. Within 24–72 hours post-NaCl exposure, plants respond to near-toxic Na^+^ levels, progressively activating mechanisms like Na^+^ exclusion and tissue tolerance to mitigate ionic stress (Munns and Tester, 2008). SPL02 has more expressed genes in 6 DAT than in 9 DAT, while Mo17 has more expressed genes in 9 DAT than in 6 DAT. Interestingly, by 7 DAT, plant biomass increased, indicating adaptability to continued salt exposure. Studies have shown that plants treated with salt for this duration exhibit strong recovery during the post-treatment phase, possibly due to protective mechanisms activated in response to salt exposure (Kawasaki et al., 2001; Chun et al., 2021). Additionally, increased stomatal conductance over a 7–21-day period suggests regained water regulation capacity, a critical aspect of salt stress adaptation (Li et al., 2023a).

Differences in DEGs between the salt-tolerant SPL02 and the salt-sensitive Mo17 reflect underlying genetic diversity, varying response mechanisms, baseline expression levels, and differences in stress-response prioritization. The majority of DEGs were unique to each inbred line, highlighting the role of genetic background in shaping salt stress adaptation (Wu et al., 2021). SPL02 exhibited a larger number of DEGs than Mo17, suggesting that its broader gene activation may contribute to its enhanced salt tolerance. Additionally, in both lines, more DEGs were upregulated than downregulated, indicating an overall adaptive gene expression shift in response to high-salt environments (Yoo et al., 2023). Under CK conditions, both lines displayed a greater number of DEGs than under T conditions, likely due to environmental and developmental cues that drive a wide range of gene expression (Sicilia et al., 2019). Over the course of salt exposure, a distinct divergence emerged: SPL02’s DEG count decreased as it achieved homeostasis, while Mo17’s increased, suggesting continued difficulty in stabilizing cellular processes. SPL02’s early activation of stress responses may facilitate a stabilized gene expression profile over time, while Mo17, with delayed activation, continued to increase DEG expression as it struggled to cope with ongoing stress.

### Functional annotation of the DEG expression patterns under salt stress

PPI analysis provided further insights, identifying six main protein groups critical for signal transduction and adaptive stress responses. The first group, including sodium/hydrogen exchangers and ammonium transporters, is key for intracellular ion regulation, essential for cellular osmoregulation and mitigation of ion toxicity (Zhu, 2001; Maathuis et al., 2014). The second group is involved in abscisic acid (ABA) signaling, which coordinates responses to both drought and salt stress. Interacting with phosphatases that modulate ABA receptor activity, these proteins enhance downstream stress responses and promote salt tolerance (Park et al., 2009; Fujita et al., 2011). The third group, containing phosphoenolpyruvate carboxykinase homologs (Pck2 and Pck1), supports gluconeogenesis, aiding energy production and metabolic balance under stress conditions to sustain physiological functions (Walker et al., 2014). The fourth group consists of calcium-signaling proteins, which act as secondary messengers under salt stress. This interaction supports calcium-dependent signaling processes, which are essential for activating stress-responsive genes and proteins (Reddy et al., 2011). The fifth group includes RNA-binding proteins, such as pre-mRNA-splicing factor prp45, and proteins with RNA-recognition motifs (RRM/RBD/RNP), involved in post-transcriptional regulation processes like mRNA splicing, stability, and translation, allowing precise gene expression adjustments in response to salt stress and ensuring the production of key stress-response proteins (Glisovic et al., 2008). Finally, ethylene-signaling proteins make up the sixth group, contributing to a hormone-driven pathway that modulates responses to environmental stress. These interactions suggest a synergistic role in enhancing salt tolerance (Kazan, 2015). Together, these protein groups underscore the complexity of maize’s response to salt stress, in which multiple signaling pathways work in concert to enable resilience in high-salt conditions. Each group contributes unique functions that highlight promising targets for genetic and biotechnological interventions aimed at improving maize’s salt tolerance—particularly through pathways involved in ion regulation, ABA and ethylene signaling, and metabolic adjustments. The findings of this study thus establish a foundation for future research to explore the molecular basis of salt tolerance and guide the development of salt-tolerant maize varieties, supporting agricultural productivity in increasingly saline environments.

The functional annotation of DEGs provides valuable insights into the molecular mechanisms underlying salt stress responses in maize (Li et al., 2017). Under CK conditions, SPL02 showed significant enrichment in KEGG pathways involved in secondary metabolite biosynthesis, carbon metabolism, and glycolysis/gluconeogenesis; Mo17 showed significant enrichment in KEGG pathways related to secondary metabolite biosynthesis, amino acid metabolism, and MAPK signaling. These metabolic activities may support basic growth and physiological functions, enhancing the vitality of plants under non-stressed conditions (Reshi et al., 2023). Under T conditions, SPL02 showed significant enrichment in pathways involved in secondary metabolites, MAPK signaling, and phenylpropanoid biosynthesis. Similarly, Mo17 showed significant enrichment in secondary metabolite biosynthesis and metabolic pathways. The activation of these pathways indicates a shift toward metabolic processes that facilitate the production of osmoprotectants, antioxidants, and signaling molecules, all critical for building salt tolerance (Wu et al., 2021; Jia et al., 2022; Sun et al., 2024).

GO enrichment analysis further highlighted the dynamic changes in biological processes associated with salt stress response in two maize inbred lines. Under CK conditions, SPL02 showed enrichment in signal transduction and organic metabolic processes, reflecting normal growth and functions (Shiade et al., 2024). Under T conditions, however, SPL02 associated with abiotic stimulus responses, hormone signaling, and osmotic stress responses became more prominent, signaling a shift in plant physiological processes to manage salt stress. These hormone-related changes play a key role in adapting to environmental challenges, affecting both growth and stress resilience (Yue et al., 2022). Salt treatment can cause changes in osmotic pressure within plant cells, affecting cellular water balance and ion homeostasis, ultimately impacting plant growth and development (Zhu, 2016). Osmotic stress, in particular, exerts a strong effect on growth rates during the initial stages of salt exposure, sometimes more significantly than ionic toxicity (Ren et al., 2020). Mo17 exhibited similar trends, with enrichment in genes related to osmotic stress response, hormone signaling, and salt stress responses under T conditions, suggesting a shared activation of certain stress-responsive pathways across both inbred lines, despite specific differences in genotype response.

Furthermore, the identification of 597 shared, highly expressed DEGs under T conditions across both SPL02 and Mo17 highlights their likely significance in maize’s salt tolerance mechanisms. These shared DEGs likely form core components of the salt stress signaling pathways. When confronted with salt stress, plants activate several adaptive mechanisms, such as osmotic regulation, ion exclusion, and antioxidant defenses, to mitigate harmful effects (Zhu, 2001). DEGs involved in ion transport, osmotic control, and stress signaling are key to understanding the differences in salt tolerance observed across different maize genotypes (Shabala and Cuin, 2008). The enrichment of GO terms associated with abiotic stress, osmotic stress, and defense responses emphasizes the critical role of these genes in orchestrating an adaptive response to salt conditions.

### Identification of key gene modules associated with salt stress response in maize using WGCNA

Applying WGCNA to examine maize’s response to salt stress has yielded significant insights into the genetic framework of stress tolerance. By analyzing 597 candidate genes identified in the T group, we were able to uncover gene modules with synergistic expression patterns, shedding light on key gene networks. The DEGs within the blue module particularly highlight its importance in salt stress adaptation.

Notably, the ME expression in the blue module increased in Mo17 but decreased in SPL02 at 6 DAT. This differential expression pattern may reflect each line’s unique adaptive strategies under prolonged salt stress. GO enrichment analysis identified several reactions and signaling pathways that play crucial roles in plant adaptation to salt stress. When maize is exposed to high salinity, defense mechanisms are activated, involving metabolic, gene expression, and physiological adjustments to enhance stress resilience (Ding et al., 2009). Osmotic stress notably affects the physiological function of maize roots, often limiting root growth and adversely impacting overall plant development (Hu et al., 2022). Additionally, salt stress induces water deficiency responses, including reduced growth and altered stomatal morphology in leaves (Li et al., 2022a). In response, maize releases ABA, a hormone crucial for stress resistance, which helps mitigate the detrimental effects of salt stress and maintains growth stability (Li et al., 2024). Adaptation to high-salinity environments requires further regulation of ion balance and metabolic pathways to prevent growth suppression (Ding et al., 2023). Together, these mechanisms underscore the plant’s ability to adjust physiologically to salt stress.

Maize exhibits significant genetic variation in salt tolerance, with some inbred lines showing improved resistance through mechanisms like ion transport and antioxidative responses (Zhang et al., 2022; Fang et al., 2024). However, compared to crops like rice, barley, and sorghum, maize’s salt tolerance remains limited due to its less efficient salt exclusion and slower osmotic adjustment processes (Pour-Aboughadareh et al., 2021; Atta et al., 2023; Vennam et al., 2024). Rice has more advanced mechanisms, such as the regulation of sodium transport via *OsHKT1;5* and *OsSOS1* and the accumulation of compatible solutes like glycine betaine, offering higher salt tolerance (Al Nayef et al., 2020; Ponce et al., 2021). Barley and sorghum, both more tolerant than maize, use genes like *HvHKT1* and *HvSOS1* for sodium ion regulation and better root architecture to manage salt stress (Thabet and Alqudah, 2023; Peduzzi et al., 2024). While maize shows promise for salt tolerance improvement through breeding, it still lags behind other crops in its overall ability to cope with high salinity (Vennam et al., 2024). The identification of key genes and pathways involved in salt tolerance provides valuable insights for improving maize’s resilience to saline environments. In this study, we identified five hub genes: *Zm00014ba252960*, *Zm00014ba364880*, *Zm00014ba260500*, *Zm00014ba233550*, and *Zm00014ba293420*. These gene annotations suggest that they play crucial roles in maize’s salt stress response. Phosphate import ATP-binding protein PstB1 indirectly supports stress signaling by ensuring phosphate supply (Poirier and Bucher, 2002). Phosphate is essential for energy metabolism and cellular functions during salt stress, and the upregulation of transporters like PstB1 helps maintain nutrient balance and enhances stress resilience (Kawa et al., 2016). *Zm00014ba364880* Glycosyltransferases help protect cells from salt-induced osmotic stress by glycosylating compounds and modifying cell wall components, which supports cellular integrity. They are also involved in regulating plant hormone synthesis and metabolism, contributing to adaptive responses to salt stress (Zhang et al., 2021a). Studies have demonstrated that the expression of the WRKY transcription factor WRKY71 is induced by salt stress, significantly influencing the physiological responses of plants. Under such stress conditions, WRKY71 enhances the plant’s salt tolerance by regulating the expression of downstream salt-responsive genes (Bakshi and Oelmüller, 2014; He et al., 2024). As a key player in the modulation of stress-responsive genes, WRKY71 orchestrates defense mechanisms that bolster the plant’s resilience to adverse environmental conditions (Rushton et al., 2010). Lipoxygenase 2.3 (chloroplastic) is involved in lipid metabolism and the production of signaling molecules like jasmonic acid, which help modulate ion homeostasis and oxidative stress during salt stress. It plays a key role in activating stress responses and aiding plant adaptation to environmental stressors (Feussner and Wasternack, 2002). Additionally, peroxisomal membrane protein 11-5 plays a crucial role in the metabolism of reactive oxygen species (ROS), which is essential for maintaining cellular redox homeostasis and protecting cells from oxidative damage (Hu et al., 2012). Together, these functional annotations and hub gene identifications provide a comprehensive view of the molecular mechanisms underlying salt stress tolerance in maize.

We have identified several KEGG pathways related to salt stress, such as MAPK signaling, phenylpropanoid biosynthesis, and hormone signaling pathways, which are crucial for maize’s response to salt stress. Five hub genes play key roles in these pathways. *Zm00014ba252960* (PstB1) is a phosphate import ATP-binding protein responsible for phosphate uptake, which influences energy status and secondary metabolism (Nogia and Pati, 2021). The availability of phosphate impacts MAPK signaling and phenylpropanoid biosynthesis, which are essential for stress responses (Yang et al., 2021). *Zm00014ba364880* (glycosyltransferase) is involved in modifying secondary metabolites, including those in the phenylpropanoid biosynthesis pathway. It adds sugar groups to metabolites, affecting their solubility, storage, and bioactivity (Gharabli et al., 2023). This enzyme’s activity is regulated by the transcription factor WRKY, which also indirectly influences MAPK signaling and hormone signaling through its modulation of glycosyltransferase activity (Miao et al., 2023). *Zm00014ba260500* (WRKY71) is a transcription factor that regulates genes in the phenylpropanoid biosynthesis pathway, producing metabolites like flavonoids and lignins, which are important for plant defense under stress (Phukan et al., 2016). WRKY is regulated by MAPK signaling, making it a central hub for integrating stress responses with metabolic processes (Luo et al., 2023; Tang et al., 2023). *Zm00014ba233550* (LOX2.3), involved in the lipoxygenase pathway, produces jasmonic acid (JA), a critical hormone in stress responses (Singh et al., 2022). MAPK signaling activates LOX2.3, which in turn drives the production of jasmonic acid, impacting defense mechanisms and stress responses (Zhao et al., 2014). *Zm00014ba293420* (PMP11-5) is involved in peroxisomal functions, such as fatty acid metabolism, ROS detoxification, and hormone metabolism. Although PMP11-5 is not directly involved in MAPK signaling, it indirectly regulates these pathways by controlling ROS levels, lipid metabolism, and hormone synthesis, particularly jasmonates, which are important for stress responses (Su et al., 2019; Sandalio et al., 2021). These interactions may be indirect and are part of a complex network of signaling and metabolism, where hormones, nutritional status, stress signals, and transcription factors converge. Further experimental evidence would be needed to clarify these potential indirect interactions. The roles of these genes in processes such as phosphate transport, cell wall modification, transcriptional regulation, lipid signaling, and ROS metabolism underscore the intricate network of responses that enable maize to manage salt stress effectively. This knowledge expands our understanding of the genetic and biochemical basis for salt tolerance and identifies potential targets for future research aimed at developing salt-tolerant maize varieties through genetic or biotechnological interventions. The qRT-PCR analysis results provide robust evidence for the differential expression of these genes under salt stress, further confirming their potential roles in maize’s salt tolerance mechanisms. Identifying hub genes linked to salt tolerance bridges genomic discoveries and the development of salt-tolerant maize varieties. These genes can be incorporated into marker-assisted selection (MAS) to expedite breeding efforts for enhanced salinity resilience. Specifically, genes such as PstB1, WRKY71, and lipoxygenase 2.3 can be targeted through gene editing and transgenic approaches to further bolster salt tolerance, expanding the genetic pool. Combining modern techniques with traditional breeding enables more efficient development of resilient maize varieties with stable yields, ensuring broad applicability in breeding programs.

To further advance the application of these findings, future research could focus on functional validation studies for the identified hub genes. Investigating their role in salt tolerance through transgenic maize lines would provide a more comprehensive understanding of their specific contributions. Additionally, exploring the interactions between these genes and environmental factors such as soil composition or soil water salinity would provide deeper insights into the molecular mechanisms underlying salt tolerance. Such studies could pave the way for more targeted breeding strategies to enhance crop resilience under saline conditions.

## Conclusion

This study provides a comprehensive analysis of salt stress responses in the seedling stage of two maize inbred lines, SPL02 and Mo17. Transcriptomic analysis identified 8,971 DEGs, which were organized into seven clusters, each enriched in pathways relevant to salt tolerance. GO enrichment analysis highlighted processes involved in abiotic stress response, hormone signaling, osmotic regulation, and abscisic acid signaling, while KEGG pathway analysis revealed roles for secondary metabolite biosynthesis, carbon metabolism, MAPK signaling, and phenylpropanoid biosynthesis. These pathways suggest essential roles in maize’s adaptive response to salt stress. A further WGCNA of 597 selected genes identified five modules, with the blue module showing a positive correlation with salt tolerance traits. The hub genes in this module, including PstB1, glycosyltransferase, WRKY71, lipoxygenase 2.3, and peroxisomal membrane protein 11-5, were identified as potential regulators in maize’s salt stress response. Our findings provide insights into the molecular mechanisms of salt tolerance, identifying hub genes and pathways that may guide breeding strategies to enhance salt resilience in maize cultivars.

## Data Availability

The datasets presented in this study can be found in online repositories. The names of the repository/repositories and accession number can be found below: https://ngdc.cncb.ac.cn/gsa/, CRA022274.

## Supplementary material

Supplementary Figure 1 Pearson’s correlation coefficient heatmap.

Supplementary Figure 2 Principal component analysis of identified genes.

Supplementary Figure 3 Common DEGs in SPL02 and Mo17.

Supplementary Figure 4 Common DEGs under salt treatment in SPL02 and Mo17.

Supplementary Figure 5 Molecular function of common DEGs under salt treatment in SPL02 and Mo17.

Supplementary Figure 6 Molecular function of blue module.

Supplementary Table 1 Sequencing data preprocessing results.

Supplementary Table 2 Protein-protein interactions information of DEGs.

Supplementary Table 3 Primers used in this qRT-PCR experiment.

Supplementary Table 4 GO terms and KEGG pathway annotations of hub genes.

Supplementary Table 5 Protein-protein interactions information of top 10 KEGG pathways in blue module.
