# Supplementary figures and images for: Dynamic molecular regulation of salt stress responses in maize (Zea mays L.) seedlings
Source: Front Plant Sci. 2025 Feb 25;16:1535943. doi: 10.3389/fpls.2025.1535943 (PMC11893837; doi:10.3389/fpls.2025.1535943)

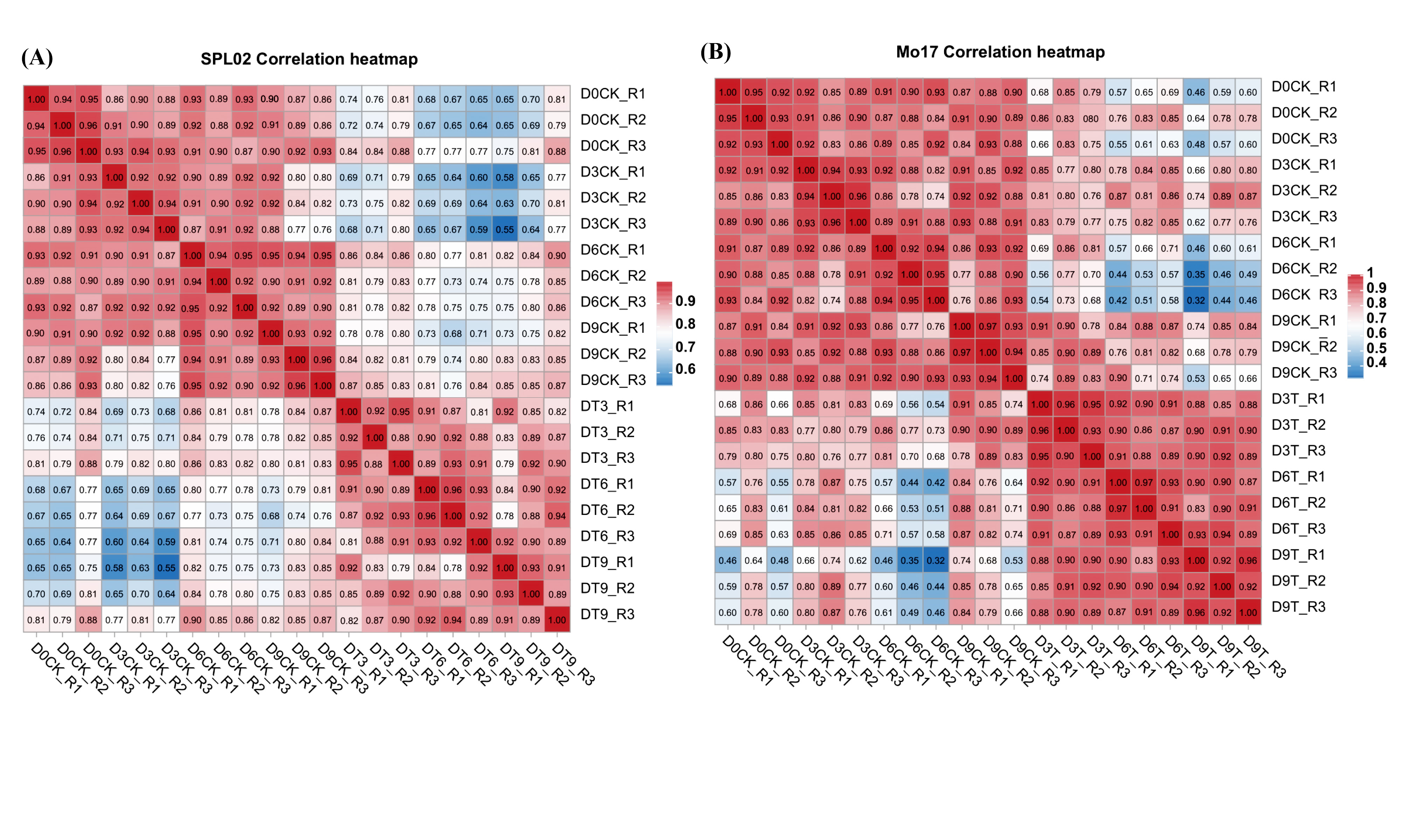

Supplement: Supplementary Figure 1 — Pearson’s correlation coefficient heatmap. [file Image1.jpeg]

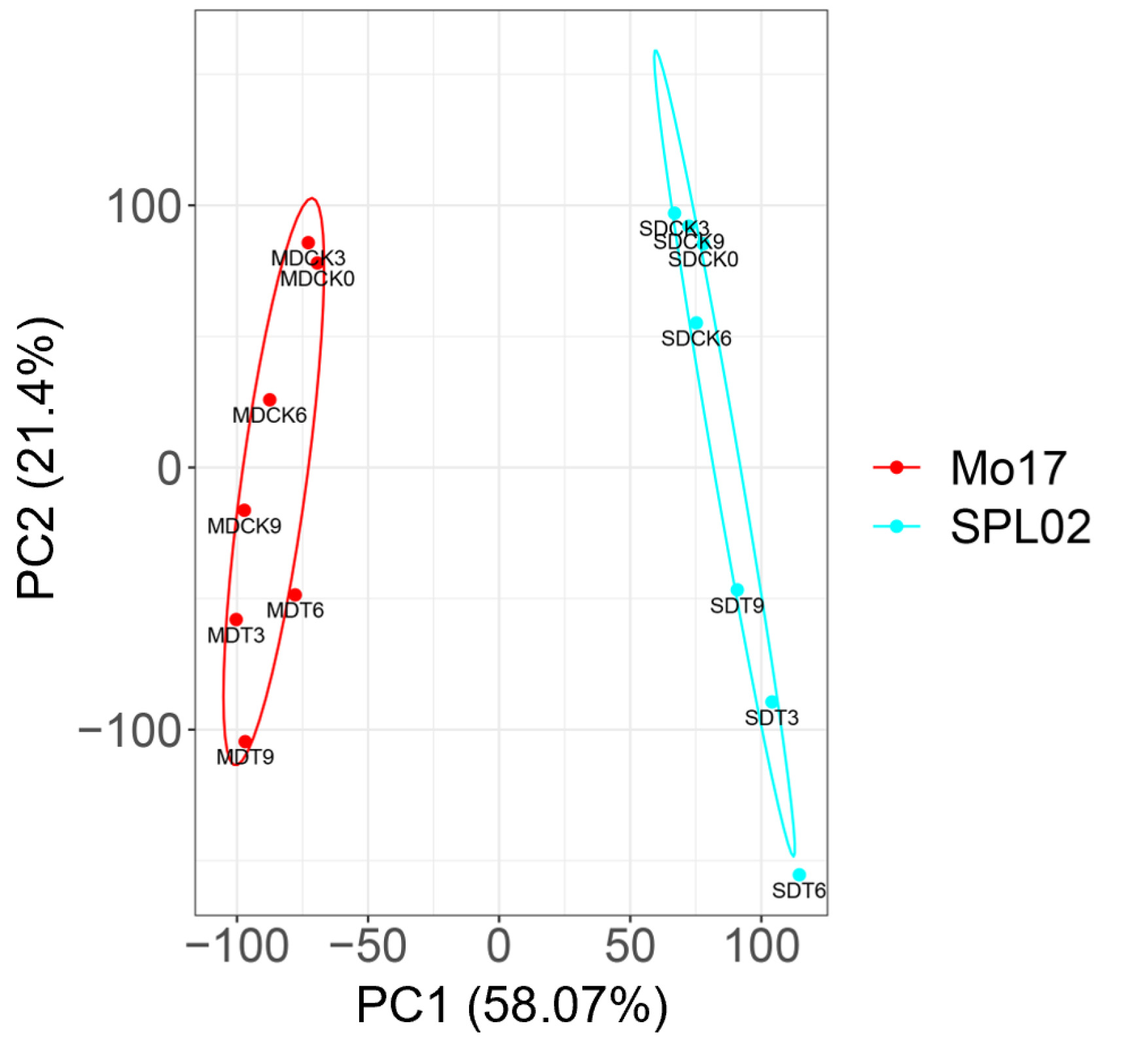

Supplement: Supplementary Figure 2 — Principal component analysis of identified genes. [file Image2.jpeg]

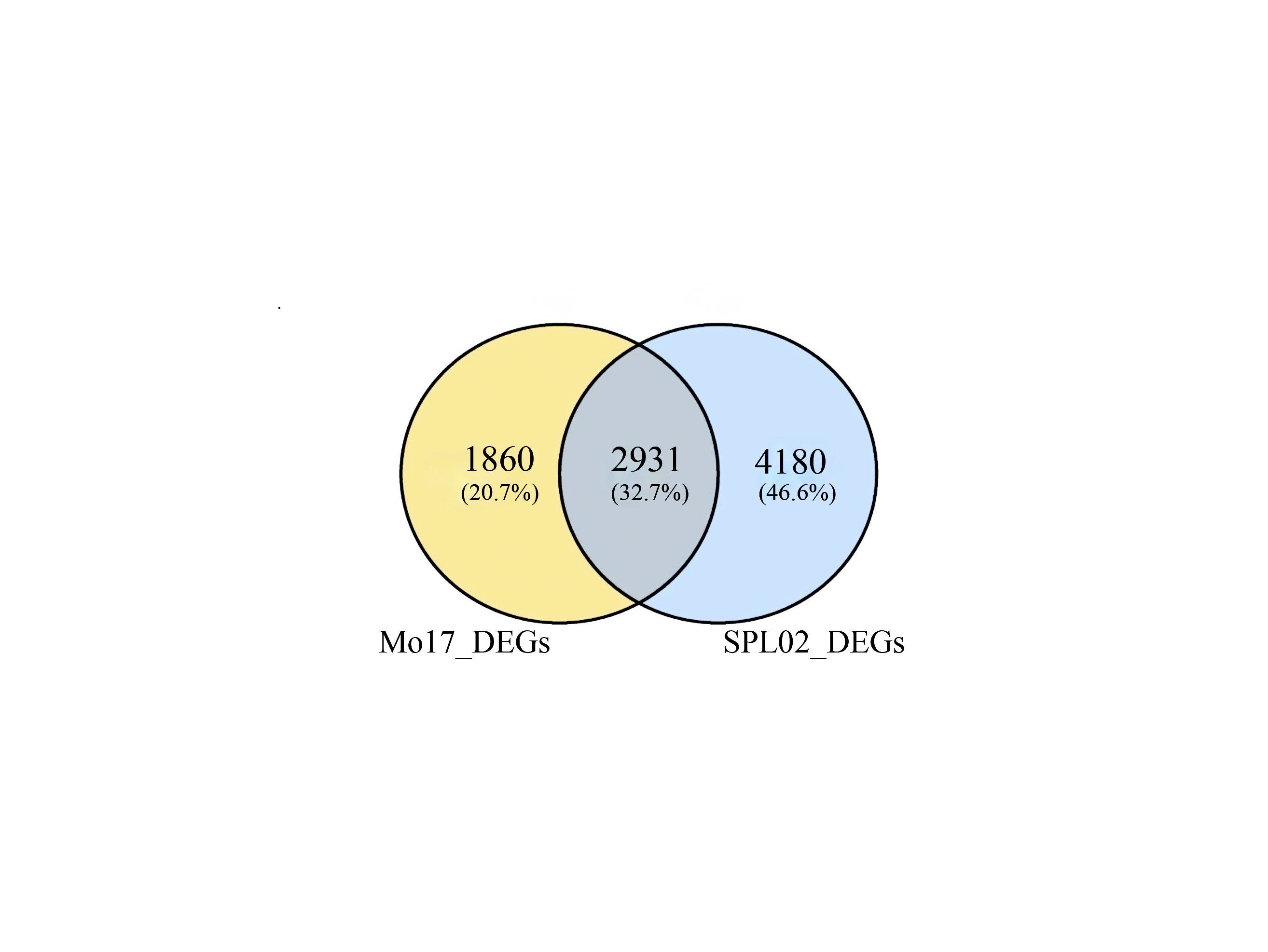

Supplement: Supplementary Figure 3 — Common DEGs in SPL02 and Mo17. [file Image3.jpeg]

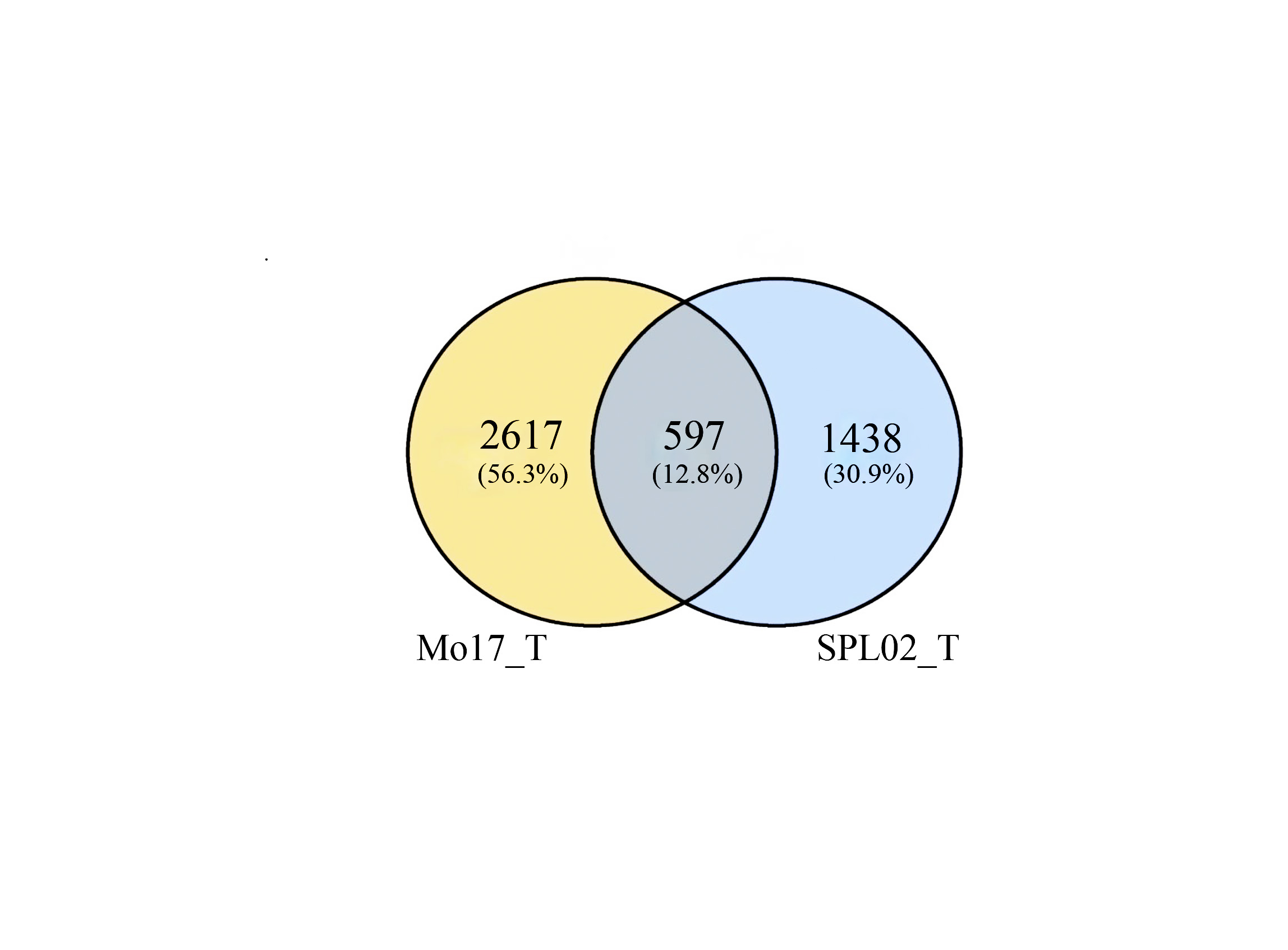

Supplement: Supplementary Figure 4 — Common DEGs under salt treatment in SPL02 and Mo17. [file Image4.jpeg]

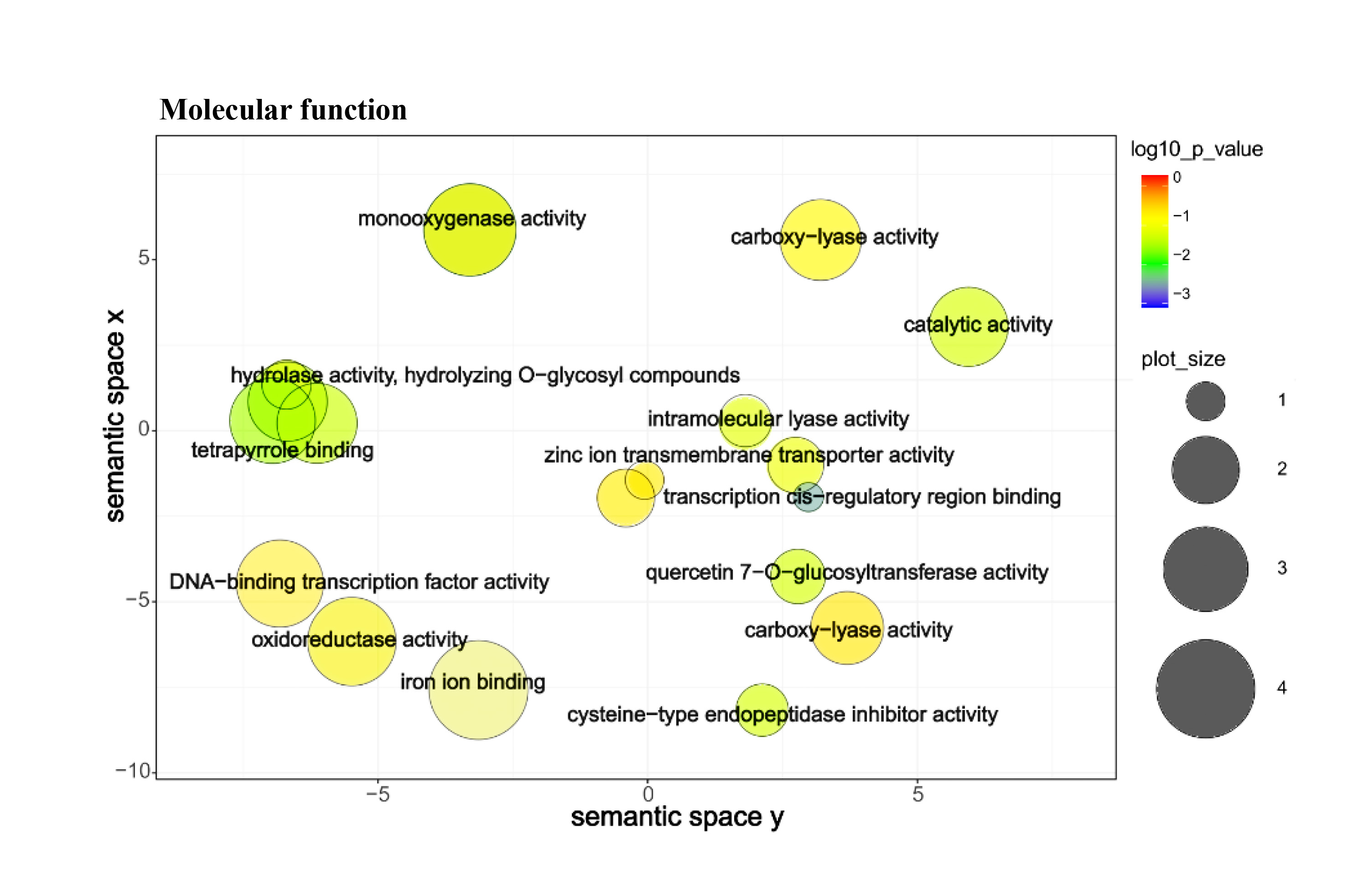

Supplement: Supplementary Figure 5 — Molecular function of common DEGs under salt treatment in SPL02 and Mo17. [file Image5.jpeg]

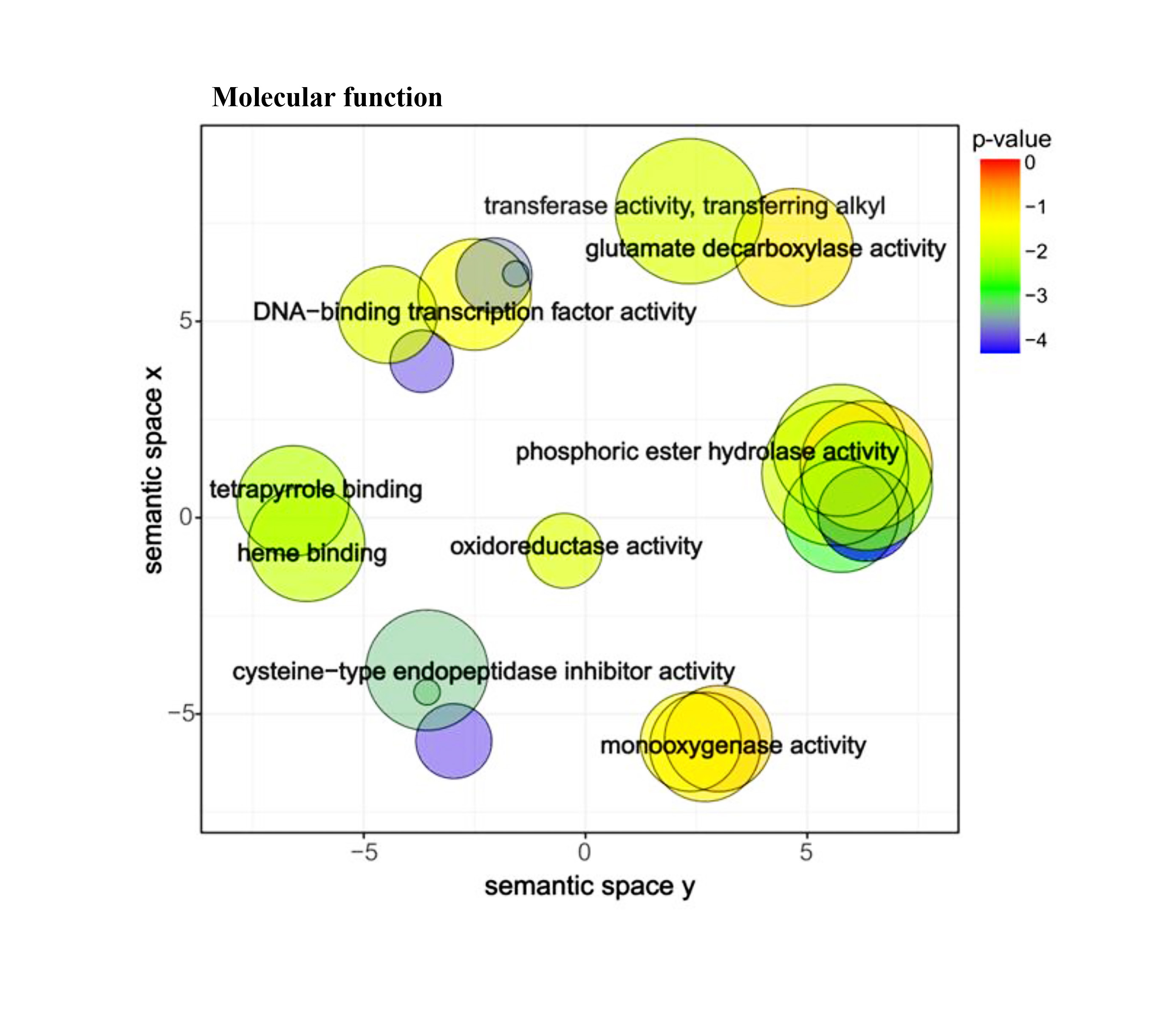

Supplement: Supplementary Figure 6 — Molecular function of blue module. [file Image6.jpeg]
